# Circular RNA *CDR1as* disrupts the p53/MDM2 complex to inhibit Gliomagenesis

**DOI:** 10.1186/s12943-020-01253-y

**Published:** 2020-09-07

**Authors:** Jiacheng Lou, Yuchao Hao, Kefeng Lin, Yizhu Lyu, Meiwei Chen, Han Wang, Deyu Zou, Xuewen Jiang, Renchun Wang, Di Jin, Eric W.-F. Lam, Shujuan Shao, Quentin Liu, Jinsong Yan, Xiang Wang, Puxiang Chen, Bo Zhang, Bilian Jin

**Affiliations:** 1 Department of Neurosurgery, The Second Affiliated Hospital; Institute of Cancer Stem Cell, Cancer Center, Dalian Medical University, Dalian, 116044 Liaoning People’s Republic of China; 2 Department of Obstetrics and Gynecology, The Second Xiangya Hospital, Central South University, Changsha, 410011 Hunan People’s Republic of China; 3 Department of Hematology, The Second Affiliated Hospital of Dalian Medical University, Dalian, 116044 Liaoning People’s Republic of China; 4 The Second Clinical Medicine School, Lanzhou University, Lanzhou, 730000 Gansu People’s Republic of China; 5 Department of Surgery and Cancer, Imperial College London, W12 0NN, London, UK; 6 Key Laboratory of Proteomics, Dalian Medical University, Dalian, 116044 Liaoning People’s Republic of China; 7 Present Address:Department of Neurosurgery, Shenzhen People’s Hospital, the Second Clinical Medical College of Jinan University, The First Affiliated Hospital of Southern University of Science and Technology, Shenzhen, 518020 Guangdong People’s Republic of China

**Keywords:** *CDR1as*, p53, MDM2, DNA damage, Glioma

## Abstract

**Background:**

Inactivation of the tumor suppressor p53 is critical for pathogenesis of glioma, in particular glioblastoma multiforme (GBM). MDM2, the main negative regulator of p53, binds to and forms a stable complex with p53 to regulate its activity. Hitherto, it is unclear whether the stability of the p53/MDM2 complex is affected by lncRNAs, in particular circular RNAs that are usually abundant and conserved, and frequently implicated in different oncogenic processes.

**Methods:**

RIP-seq and RIP-qPCR assays were performed to determine the most enriched lncRNAs (including circular RNAs) bound by p53, followed by bioinformatic assays to estimate the relevance of their expression with p53 signaling and gliomagenesis. Subsequently, the clinical significance of *CDR1as* was evaluated in the largest cohort of Chinese glioma patients from CGGA (*n* = 325), and its expression in human glioma tissues was further evaluated by RNA FISH and RT-qPCR, respectively. Assays combining RNA FISH with protein immunofluorescence were performed to determine co-localization of *CDR1as* and p53, followed by CHIRP assays to confirm RNA-protein interaction. Immunoblot assays were carried out to evaluate protein expression, p53/MDM2 interaction and p53 ubiquitination in cells in which *CDR1as* expression was manipulated. After *AGO2* or *Dicer* was knocked-down to inhibit miRNA biogenesis, effects of *CDR1as* on p53 expression, stability and activity were determined by immunoblot, RT-qPCR and luciferase reporter assays. Meanwhile, impacts of *CDR1as* on DNA damage were evaluated by flow cytometric assays and immunohistochemistry. Tumorigenicity assays were performed to determine the effects of *CDR1as* on colony formation, cell proliferation, the cell cycle and apoptosis (in vitro), and on tumor volume/weight and survival of nude mice xenografted with GBM cells (in vivo).

**Results:**

*CDR1as* is found to bind to p53 protein. *CDR1as* expression decreases with increasing glioma grade and it is a reliable independent predictor of overall survival in glioma, particularly in GBM. Through a mechanism independent of acting as a miRNA sponge, *CDR1as* stabilizes p53 protein by preventing it from ubiquitination. *CDR1as* directly interacts with the p53 DBD domain that is essential for MDM2 binding, thus disrupting the p53/MDM2 complex formation. Induced upon DNA damage, *CDR1as* may preserve p53 function and protect cells from DNA damage. Significantly, *CDR1as* inhibits tumor growth in vitro and in vivo, but has little impact in cells where p53 is absent or mutated.

**Conclusions:**

Rather than acting as a miRNA sponge, *CDR1as* functions as a tumor suppressor through binding directly to p53 at its DBD region to restrict MDM2 interaction. Thus, *CDR1as* binding disrupts the p53/MDM2 complex to prevent p53 from ubiquitination and degradation. *CDR1as* may also sense DNA damage signals and form a protective complex with p53 to preserve p53 function. Therefore, *CDR1as* depletion may play a potent role in promoting tumorigenesis through down-regulating p53 expression in glioma. Our results broaden further our understanding of the roles and mechanism of action of circular RNAs in general and *CDR1as* in particular, and can potentially open up novel therapeutic avenues for effective glioma treatment.

## Background

Glioma is the most common tumor of the central nervous system (CNS), representing 30% of all CNS tumors and 80% of all malignant brain tumors [1]. Almost all glioma relapses despite intensive treatments with surgery, radiation, and chemotherapy. Particularly, glioblastoma multiforme (GBM), the most common and most aggressive glioma, is characterized by a median overall survival that has remained static at around 15 months for decades [2], highlighting an urgent need for intensifying study of the underlying mechanisms of this lethal disease.

p53 is a master regulator of diverse anti-proliferative functions [3, 4]. It is well established that mutations and/or inactivation of p53 are critical for tumorigenesis. Malignant tumors often harbor mutations to *TP53* gene, which ultimately gives rise to the oncogenic phenotype. Glioma, particular GBM, however, usually possesses a structurally intact *TP53* gene [5]. Therefore, the augmented proliferation and resistance to cytotoxic treatment in GBM has been attributed to the loss of p53 functions by inactivation [6]. MDM2, the major negative regulator of p53, interacts with p53 to form a stable complex. When bound to p53, MDM2 not only prevents transcriptional activation of p53, but also promotes p53 degradation through ubiquitination. p53, in turn, stimulates transcription of MDM2 by binding to its promoter region. Consequently, there exists a regulatory feedback loop between p53 and MDM2 [7]. Multiple factors have been found to bind to either p53 or MDM2 to modulate the stability of the p53/MDM2 complex [8–11]. However, our understanding of how the p53/MDM2 complex is modulated during tumorigenesis remains incomplete.

Long non-coding RNAs (lncRNAs) are a diverse class of RNAs of more than 200 nt, which lack coding potential and have been demonstrated to be involved in tumorigenesis [12, 13]. Circular RNAs (circRNAs) that are covalently closed, however, are among the least-well characterized lncRNA transcripts. The biogenesis of circRNAs is abundant [14, 15], conserved [14, 15] and cell type specific [15–17], suggesting that they have a key role in regulating gene expression and cell fate [15, 18, 19]. Being dysregulated in multiple cancers, circRNAs are frequently implicated in different oncogenic processes [20–22]. The circular nature of these transcripts, however, makes their detection, quantification and functional characterization challenging. Hitherto, our knowledge of circRNAs, as well as their roles and mechanisms of action, has been very limited [21].

p53 has two distinct nucleic acid-binding domains including the core DNA-binding domain (DBD) and the C-terminal regulatory domain (CTD) [23]. It has been reported that p53 probably binds RNA molecules via its CTD rather than the core DBD, although the physiological role of this interaction remains enigmatic. It is still unclear whether p53 is able to bind to lncRNAs that are frequently involved in tumorigenesis. CircRNAs have been found to function as miRNA sponges. Recently, they have been demonstrated to play a key role in tumorigenesis through its capacity to bind proteins [24]. Hitherto, the functional significance of the interaction between p53 and circRNAs on the formation of the p53/MDM2 complex and therefore p53 stability is yet to be elucidated.

In this study, the circRNA *CDR1as* has been identified to bind physically to p53 protein at its core DBD. So far, only preliminary studies have been conducted to investigate the role of *CDR1as* in the oncogenic process, and they are mainly related to *CDR1as* functioning as a miRNA sponge. However, the results from these different studies are mutual contradictory [25–27]. *CDR1as* is abundantly expressed in mammalian brain in a conserved pattern, implying it has a critical function in brain development and disease. Consistently, the most recent work has demonstrated that *CDR1as* is essential for brain development and various brain functions [28]. Therefore, it is very plausible that *CDR1as*, when dysregulated or inactivated, may contribute to tumorigenesis of glioma, the most common and yet incurable tumor of CNS.

In the present study, we verify that *CDR1as* is significantly down-regulated in glioma, particular in GBM, and its expression is positively correlated with patient prognosis. We demonstrate that *CDR1as* inhibits tumor growth both in vitro and in vivo. Rather than being a miRNA sponge, *CDR1as* functions through binding tightly to p53 within its DBD region. The p53 DBD that is essential for ubiquitination and forms a crucial interface for the MDM2 interaction. Thus, *CDR1as* binding may disrupt the p53/MDM2 complex. Consequently, this circRNA molecule may promote p53 protein stability through restricting its ubiquitination. Binding firmly to p53, *CDR1as* may sense signals of DNA damage and serve to protect p53 function. Therefore, inactivation of *CDR1as* contributes significantly to tumorigenesis of glioma.

## Methods

### Plasmids and antibodies

Plasmid of pGL4-p53 was purchased from Promega. The Plasmids of pCDNA3.1-*CDR1as* was a gift of Dr. Nikolaus Rajewsky. Plasmids of *Myc-p53*, *Myc-MDM2*, *PCDH-p53* and *HA-Ub* were a gift of Dr. Zhang Lingqiang. The pCDNA3.1(+) circRNA mini vector was purchased from Addgene (60648). The plasmid of *HA-MDM2* was purchased from Sino Biological (HG11206-CY). All expression constructs were verified by DNA Sequencing.

Antibodies against p53 (DO-1) (ab1101), MDM2 (ab3110), or PUMA (ab33906) were purchased from Abcam Inc. The p53 polyclonal antibody (FL393) and the Myc antibody (9E10), and the HA antibody (sc7392) were purchased from Santa Cruz Biotechnology. The antibody against Flag (M2) was purchased from Sigma. The antibody against GAPDH (AC027) and p21 (A11877) were purchased from Abclonal. The antibody against Phospho-Histone H2A.X (Ser139) (2577 s) was purchased from CST.

### RNA Immunoprecipitation (RIP) assays

After crosslinking with 0.5% formaldehyde for 10 min at room temperature, cells were harvested and lysed in RIP lysis buffer (25 mM Tris-HCl pH 7.4, 150 mM NaCl, 1 mM EDTA, 1% NP-40 and 5% glycerol) with RNasin (1000 U/ml), DNase I (50 U/ml) and protease inhibitor cocktail. After the genomic DNA was digested, lysates were further subjected to sonication. Supernatants cleared by centrifugation were incubated with the anti-p53 antibody or IgG overnight at 4 °C. Protein A/G beads were added for a further 4 h incubation at room temperature. After the beads were washed with wash buffer (25 mM Tris-HCl pH 7.4, 500 mM NaCl, 1 mM EDTA, 1% NP-40 and 5% glycerol), immunocomplexes of p53 and RNAs were de-crosslinked at 95 °C for 15 min. The immunoprecipitated RNAs were then purified using Trizol and ethanol precipitation, and subjected to qPCR analysis (Additional file 1: Table S2.) or high-throughput sequencing using PacBio Sequencing.

### Chromatin isolation by RNA purification (CHIRP) assays

The CHIRP assay [29] was carried out to verify the interaction between *CDR1as* and the p53 protein. First, tiling DNA oligonucleotide probes targeting the full-length sequence of *CDR1as* were designed using the online probe designer at singlemoleculefish.com, and 12 biotinylated tiling probes were synthesized by Takara. One potential source of noise in CHIRP is the precipitation of non-specific DNA fragments by the oligonucleotide probes. In order to circumvent this problem, we took a “split-probe” strategy as Chu C, et al. [29, 30], where we ranked all probes based on their relative positions along the target RNA and split them into two pools, with the “even” pool contains all probes numbering 2, 4, 6, etc. and the “odd” pool contains all probes numbering 1, 3, 5, etc. The pools of probes were diluted to 100 μM and stored at − 20 °C. After crosslinking with 3% formaldehyde at room temperature for 15 min, cells were lysed and chromatin collected. Subsequently, chromatin was hybridized with different pools of tiling probes of *CDR1as* as indicated at 37 °C for 4 h with shaking. Then complexes were incubated with beads conjugated with Streptavidin at 37 °C For 4 h. The beads-bound chromatin was then eluted for Western blot assays to determine the abundance of p53 protein bound by *CDR1as*.

### RNA isolation and RT-qPCR

The total RNA was extracted from tissues or cultured cells with Trizol reagent (Invitrogen) and reverse transcribed with PrimeScript RT Reagent Kit with gDNA Eraser (TaKaRa). Quantitative PCR was performed with SYBR green (SYBR Premix Ex Taq, TaKaRa) `at Agilent Mx3005P real-time PCR system (Agilent Technologies). Expression analysis in different cell types was performed using specific primers for each gene.

### Immunoblot assays

Cells were lysed in RIPA buffer with protease inhibitor cocktail. Total proteins were separated by SDS-PAGE and then transferred to nitrocellulose membranes (Millipore HATF00010). Membranes were incubated with the indicated primary antibodies overnight at 4 °C. Immunocomplexes were detected by chemiluminescence (K-12045-D50, Advansta) after incubation with the appropriate horseradish peroxidase-conjugated antibodies for 1 h.

### Immunoprecipitation (IP) assays

Cells were lysed in IP buffer with protease inhibitor cocktail. After sonication, the lysates were cleared by centrifugation at 4 °C. After incubating with primary antibodies at 4 °C overnight, protein A/G-beads were added to the lysates. Finally, immunocomplexes eluted from beads were detected using indicated antibodies by western blotting.

### Cell culture

HCT116 ^p53+/+^ and HCT116^p53−/−^ cells were cultured in McCoy’s 5A supplemented with 10% fetal bovine serum (Gibco), U87MG cell was cultured in EMEM supplemented with 10% fetal bovine serum (Gibco), MEF^p53−/−MDM2−/−^ cell was cultured in DMEM supplemented with 10% fetal bovine serum (Gibco) and LN229 was cultured in DMEM supplemented with 5% fetal bovine serum (Gibco), then maintained at 37 °C in a humidified atmosphere of 5% CO_2_.

Transfection of cells with plasmids or siRNAs (Table S2) was carried out using lipofactamine 2000 according to the manufacture’s instructions. Doxorubicin (DOXO) (S1208 Selleck) or VP16 (S1225 Selleck) was used to induce DNA damage, and their final concentrations were 400 nM and 20 μM, respectively.

### Luciferase reporter assays for p53

The plasmid encoding luciferase reporter gene for p53 was purchased from Promega (pGL4.38[luc2P/p53 RE/Hygro] Vector, E365A). The reporter plasmid was transfected into cells in 12-well plates together with siRNAs against *CDR1as* or control, or together with pcDNA3.1 encoding *CDR1as* or blank pcDNA3.1 plasmid. Forty-eight hours after transfection, cells were harvested and analyzed using a luminometer. Firefly activity was normalized to Renilla luciferase activity. Data represented means +/− SEM of three independent experiments.

### Assays of RNA fluorescence in situ hybridization (FISH) and protein immunofluorescence (IF)

To determine the abundance and positioning of *CDR1as* in tissues and cells, RNA FISH assays were performed as described by Cui et al [31] Briefly, the DNA probe targeting the end-to-head junction of *CDR1as* was labeled with FITC. After fixing in a 4% (wt/vol) paraformaldehyde solution, samples were rinsed in 1 × PBS, permeabilized in 1 × PBS with 0.5% (vol/vol) Triton X-100 (10 min), washed in 1 × PBS with 0.1% (vol/vol) Tween-20 (1 min). Probes were mixed with pre-made hybridization buffer, and then samples were incubated in hybridization buffer at 37 °C overnight. After washed with hybridization buffer at 37 °C for 15 min and quickly rinsed at room temperature three times, cells were stained in DAPI stain solution. Finally, images were taken under immunofluorescence microscope (Leica, TCS SP5II).

For protein IF assays, cells were cultured on glass slides in petri dishes. When nearly confluent, cells were fixed with 4% paraformaldehyde for 10 min, permeabilized in 0.5% Trion-X-100 for 15 min and blocked in 3% BSA for 30 min at room temperature. Then cells were incubated with the primary antibody against p53 or Phospho-Histone H2A.X overnight at 4 °C, followed by incubation with the secondary antibody conjugated with FITC for 45 min at 37 °C. Immediately after DAPI was added, images were taken with immunofluorescence microscope (Leica, TCS SP5II).

### Deubiquitination of p53

Cells were transfected with indicated constructs. Twenty-four hours after transfection, they were treated with the proteasome inhibitor MG132 (20 mM) for 4 h. The cells are harvested and lysed in the lysis buffer. Ubiquitinated p53 in lysates was immunoprecipitated with p53 (FL-393) antibody and immunoblotted with anti-HA antibody or anti-p53 antibody.

### Tumorigenic assays of *CDR1as* in vivo

Female athymic BALB/c nude mice that aged 4 weeks were randomly classified into indicated groups (*n* = 10). One million of indicated cells were subcutaneously injected into the left anterior side or the right anterior side of each mouse. Xenograft tumor growth was examined by caliper measurement. At 4 weeks post-injection, all mice were sacrificed, and tumors removed for weight measurement. Meanwhile, Kaplan-Meier assays were carried out to compare overall survival of mice between indicated groups, each of which consisting of 10 mice.

All animal studies were carried out in accordance with the National Institute of Health Guide for the Care and Use of Laboratory Animals under the approval of the SPF Laboratory Animal Center at Dalian Medical University. The protocol was approved by the Animal Care and Ethics Committee of Dalian Medical University. All surgery was performed under sodium pentobarbital anesthesia, and all efforts are made to minimize suffering in mice.

### Immunohistochemistry (IHC)

Mouse Tumor tissues fixed with 4% paraformaldehyde for 4 h at room temperature and paraffin sections (6 μm) were used for IHC analysis. Paraformaldehyde-fixed, paraffin-embedded sections were processed and stained by H&E, PCNA antibody and p53 antibody, respectively.

### Cytoplasmic and nuclear protein fractionation

U87MG cells were transfected with siNC or siCDR1as. After 48 h, the cells were treated with MG132 (20 μM) for 4 h. Then the cells were collected for fractionation analysis. Following the manufacture’s procedures, cytoplasmic and nuclear fractions were separated using NE-PER Nuclear and Cytoplasmic Extraction Reagent (Sigma, P-2714). Fractionation efficiency was validated by Western blot using antibodies specific to marker proteins for each fraction: GAPDH for cytoplasm and Histone 3 for nucleus.

### Cell proliferation and colony formation assays

Cell proliferation was monitored using CCK8 Kit (Dojindo, Rockville, MD, USA). Cells transfected with pCDNA3.1-*CDR1as*/siCDR1as or their corresponding controls (3000/well) were allowed to grow in 96-well plates. Cell proliferation was measured every 24 h following the manufacturer’s protocol. All experiments were performed in triplicates.

Colony formation was assessed by plating 1 × 10^3^ cells in 35-mm dishes. After 14 days of cultivation, crystal violet staining was used to visualize clones. Clones were counted under a light microscope and those 2 mm or greater in size were scored.

### Flow cytometric assays of cell cycle and apoptosis

To estimate the proportions of cells in different phases of the cell cycle, cellular DNA contents were measured by flow cytometry. After treatment, cells were harvested, washed twice with cold PBS, and then fixed overnight at − 20 °C in 70% ethanol. Immediately before flow cytometry, the cells were re-suspended in PBS containing PI (50 mg/ml) and DNase-free RNase (10 mg/ml). Flow cytometry was performed using a FACScalibur (Becton Dickinson, San Diego, CA, USA) system with CELL quest software. The percentages of cells in different phases of the cell cycle were determined using the FlowJo software (Tree Star Inc.).

For apoptosis analysis, the Annexin V Apoptosis Detection kit was used. The cells surface staining was performed as described above followed by being washed, re-suspended in Binding Buffer at 10^6^ cells/ml. Then the cells were incubated 10 min with Annexin V at the room temperature and protected from light. After incubation, the cells were washed, and re-suspended in 200 μl Binding Buffer. PI was added before being analyzed by flow cytometry. Data were acquired on a FACScalibur (Becton Dickinson, San Diego, CA, USA) system with CELL quest software. All analysis was performed using FlowJo software (Tree Star Inc.). The detection was repeated three times.

### RNA sequencing and bioinformatics analysis

For RIP-seq, the immunoprecipitated RNA was subjected to high-throughput sequencing using Illumina HiSeq 2500 by Novegene (Tianjin, China). Strand-specific library was constructed by using a VAHTS Total RNA-seq (H/M/R) Library Prep Kit from Illumina according to the manufacturer’s instructions. Ribosome RNAs were removed. Second-strand cDNA was synthesized using DNA polymerase I, RNase H, dNTP (dUTP instead of dTTP) and buffer. Analysis of Illumina libraries was performed using a combination of programs including STAR [32]. Raw data were first processed through perl scripts to obtain clean data by removing reads containing adaptor, ploy-N and with low quality. Reference genome and gene model annotation files were downloaded from genome website browser GENCODE directly. Paired-end clean reads were aligned to the reference genome using STAR 2.6.1 [32]. All fragment quantifications were computed using STAR setting “--quantMode GeneCounts. Differential mRNA abundance analysis was carried out with DESeq2 (http://www.r-project.org/). Genes with reads < 5 in more samples than the sample size of one of the groups being compared were pre-filtered from the analysis. We constructed heat maps by the normalized gene expression and made gene mapping loci picture via R package “Gviz”. The normalized gene expression was subjected to Gene Set Variation Analysis (GSVA), which is a non-parametric, unsupervised method for estimating variation of gene set enrichment through the samples of an expression data set.

In the CGGA dataset, we collected transcriptome sequencing data of 325 samples generated by Illumina HiSeq platform. Clinical and molecular information was obtained from the CGGA database (http://www.cgga.org.cn/). Pan-cancer and Genotype-Tissue Expression (GTEx) RNA-seq data were obtained from the TCGA in UCSC Xena platform [33]. Clinical information and data of molecular biomarkers (1p/19q co-deletion, MGMT promoter methylation, Grades) were generated from TCGA publications. Normal expression profiling of *CDR1as* in different tissues were analyzed using GEPIA as described by Tang et al [34] R package limma was used for differential gene expression analysis. R package survival was used for overall survival analysis. Cox proportional hazard model is executed by the function survival and survminer from R packages. The best-scanned cutoff points are defined as the one with the most significant (log-rank test) split. R package survival ROC was used for Receiver Operating Characteristic curve (ROC) and Area Under Curve (AUC) plotted for different durations of survival analysis.

### Statistical analysis

Student’s t-test (two-tailed), t-test with Welch’s correction, F-test were performed to analyze the data using GraphPad Prism 8.0 software and R (version 3.6.0, https://www.r-project.org/). The methods of t-test analysis in current study are as follows: the data of two groups for comparison were analyzed by F-test firstly (homogeneity test of variance). If the value of F-test > 0.05, the value of t-test was obtained according to heteroscedasticity double sample test. If the value of F-test < 0.05, the value of t-test was obtained according to the heteroscedasticity double sample test; the value of t-test < 0.05 indicated that there was significant difference between the two experimental groups. Correlations between continuous variables were evaluated by Spearman correlation analysis. The Student t-test, one-way ANOVA, and Pearson’s Chi-squared test were used to assess differences in variables between groups. The survival distributions were described by the Kaplan–Meier survival curve and the log-rank test was used to test the statistical significance. The prognostic value of *CDR1as* was estimated by Univariate and multivariate Cox proportional hazard model analysis. For most of the in vitro and animal experiments, Student’s t-tests were used to calculate the *p*-value. *p*-Values less than 0.05 were considered statistically significant. ns, no significance; *p < 0.05; **p < 0.01; ***p < 0.001; ****p < 0.0001.

## Results

### The circRNA *CDR1as* interacts with p53 and is a reliable predictor of prognosis in glioma

The tumor suppressor p53 is a master transcription factor that regulates a broad range of important functions, including DNA repair, metabolism, cell cycle arrest, apoptosis and senescence [35]. p53 is one of the most studied molecules of all time, but our understanding of its regulatory and signaling network remains incomplete. p53 has been reported to be able to bind RNA molecules, which may reversely regulate p53 activity. lncRNAs play a key role in multiple types of cancer including glioma. It is, however, far from clear whether and how p53 interacts with lncRNAs, particularly with circRNAs.

**Fig. 1:**
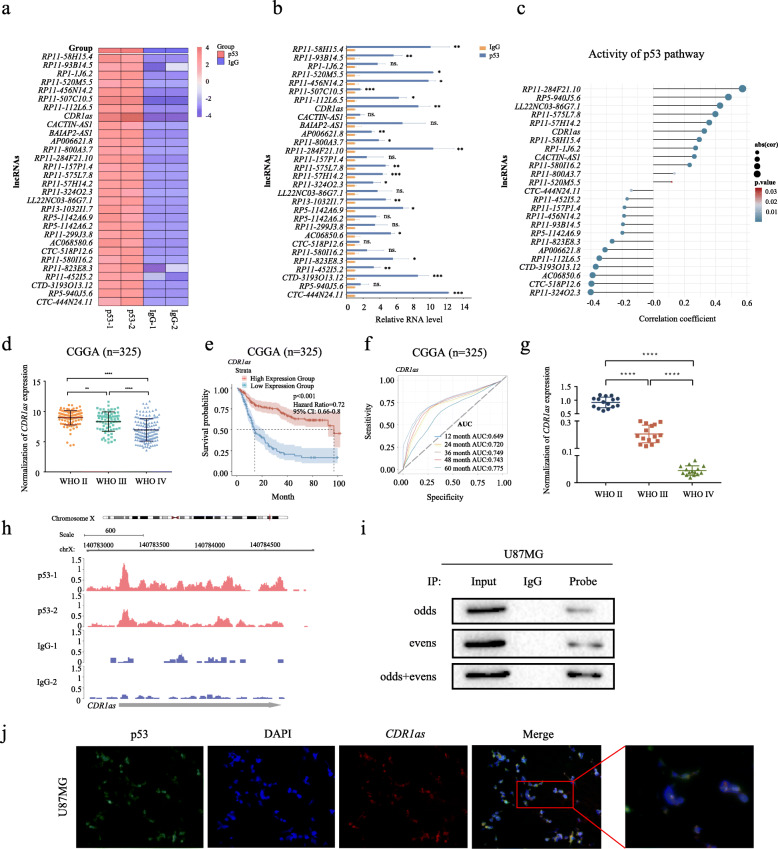
p53 physically interacts with *CDR1as* indicating glioma prognosis. **a** Heatmap of 30 most enriched lncRNAs binding to p53 protein determined by RIP-seq. **b** Validation of 30 candidate lncRNAs binding to p53 protein by RIP-qPCR. **c** Plots of the correlation between the scores of p53 pathway gene sets and expression of candidate lncRNAs in glioma samples in the CGGA cohort. **d** *CDR1as* expression in glioma with different WHO grades in the CGGA cohort. **e**, **f** Kaplan-Meier curves of the overall survival (**e**) and ROC curves (**f**) of glioma patients in the CGGA cohort. **g** RT-qPCR assays of *CDR1as* expression in glioma samples collected by ourselves. **h** Mapping of RIP-seq reads back to genomic locus of *CDR1as*. **i** Validation of the p53-*CDR1as* interaction by CHIRP. **j** Co-localization analysis of p53 and *CDR1as* in U87MG cells using protein IF and RNA FISH assays respectively. ns, no significance; *p < 0.05; **p < 0.01; ***p < 0.001; ****p < 0.0001

Using U87MG cells of GBM (*TP53* wild type), our RIP-seq assays showed that multiple lncRNAs were captured by p53 protein. The 30 most enriched lncRNAs were shown in the heatmap (Fig. 1a) and further validated by RIP-qPCR analysis (Fig. 1b). Compared to IgG, 20 of the 30 candidate lncRNAs, including *RP11-58H15.4*, *RP11-284F21.10* and *CDR1as*, were confirmed to bind p53 appreciably with *p* value < 0.05 (Fig. 1b). Next, using Spearman correlation analysis, we explored if the expression of the candidates is relevant to p53 pathway activity in clinical samples from the CGGA cohort. As shown in the heatmap (Fig. 1c), 25 out of the 30 candidate lncRNAs were significantly (*p* < 0.05) associated with the p53 pathway. The horizontal axis reveals the correlation coefficient, with the size of the bubble indicating the magnitude of the coefficient. For example, *RP11-284F21.10* expression was significantly positively associated with the activity of p53 pathway (**p < 0.01), while *RP11-324O2.3* was negatively correlated with the p53 pathway (**p < 0.01). *RP5-940J5.6* did not bind to p53 (Fig. 1b), but its expression was correlated with p53 pathway in clinical samples (**p < 0.01, Fig. 1c).

Interestingly, the circRNA *CDR1as* was found to be one of the most enriched lncRNAs binding to p53. Meanwhile, its expression was significantly correlated with p53 signaling in glioma in the CGGA cohort (Fig. 1c). These results imply that *CDR1as* may regulate p53 activity through its binding with the latter. CircRNAs are among the least well characterized lncRNA transcripts, and our knowledge of their functions in cancer is very limited. Among human tissues, *CDR1as* is most abundant in brain (Additional file 2: Figure S1A). Pan-cancer analysis showed that *CDR1as* is dysregulated in multiple types of cancer, particularly in GBM (Additional file 2: Figure S1B). Through analysis of the largest publicly available cohorts of Chinese glioma patients from CGGA (Chinese Glioma Genome Atlas) [36], we found that *CDR1as* is obviously down-regulated in glioma, in inverse correlation with WHO grades defined by histopathologic criteria (Fig. 1d). The data from CGCA showed that *CDR1as* expression is positively correlated with the overall survival of glioma patients, particularly in patients with higher grade glioma (Fig. 1e and Additional file 2: Figure S1C). Notably, *CDR1as* AUC (area under curve) from 12 to 60 months is consistently more than 0.7, suggesting that it is very reliable for predicting the hazard rate of Glioma (Fig. 1f and Additional file 2: Figure S1D). More importantly, multifactor Cox regression analysis demonstrated that *CDR1as* is an independent predictive factor for the prognosis of glioma patients (Additional file 1: Table S1).

The *CDR1as* expression pattern was further confirmed in a panel of glioma tissues (*n* = 45) collected by ourselves through RT-qPCR assays (Fig. 1g). As expected, *CDR1as* is gradually down-regulated in glioma samples along with the increase of WHO grade (Fig. 1g). Consistently, the results of the RNA FISH assays verified that *CDR1as* expression in glioma tissues (*n* = 87) is significantly lower than in normal brain tissues (*n* = 3) (Additional file 2: Figure S1E).

Further evidence demonstrated *CDR1as* binds to p53. First, the coverage tracks from the RIP-seq showed that the p53-bound RNAs cover genomic position of *CDR1as* (chromosome X, nucleotides 91, 516, 046–91, 517, 771 in GENCODE release v28) (Fig. 1h). Moreover, the CHIRP experiment confirmed the interaction between p53 and *CDR1as* using 12 probes covering the full-length sequence of *CDR1as*. All probes were ranked based on their relative positions along the target RNA and split into two pools, with the “even” pool contains all probes numbering 2, 4, 6, etc. and the “odd” pool probes numbering 1, 3, 5, etc. CircRNAs are covalently closed via back splicing machinery, lacking both 5′ and 3′ end, and are stable in vivo due to their resistance to degradation by RNaseR [37]. Compared to controls, the p53 protein is significantly enriched after *CDR1as* pull-down (Fig. 1i). Subsequently, the assays combining RNA FISH with protein IF showed that circRNA *CDR1as* is strongly co-localized with p53 protein, particularly in nucleus in GBM U87MG cells (Fig. 1j).

Collectively, we demonstrated that p53 strongly interacts with the circRNA molecular *CDR1as,* which is closely correlated to p53 signaling. These results suggested that *CDR1as* may, in turn, regulate p53 activity to modulate tumorigenesis. Being specifically abundant in brain, *CDR1as* is substantially down-regulated in glioma, particularly in GBM. Very significantly, *CDR1as* may serve as an independent factor to predict prognosis of glioma patients.

### *CDR1as* up-regulates expression of p53 protein rather than its mRNA

**Fig. 2:**
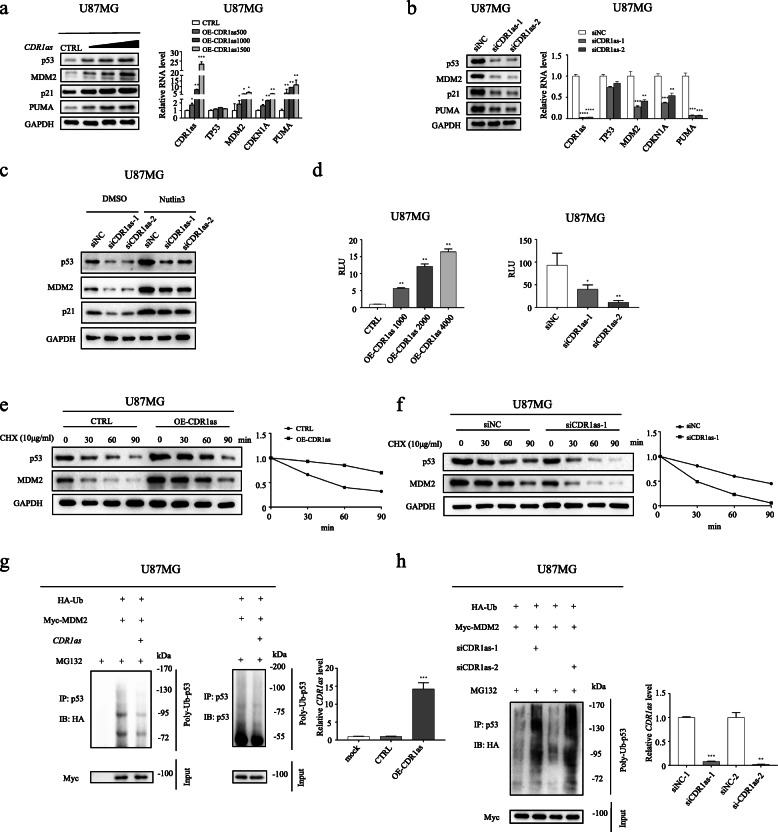
*CDR1as* up-regulates expression of p53 protein by inhibiting its ubiquitination in U87MG cells. **a** Western blot analysis of p53 and its targets (left); and validation of RNA levels of *CDR1as*, *TP53*, *MDM2*, *CDKN1A* and *PUMA* by RT-qPCR (right) in U87MG cells transfected with increasing concentrations of plasmid encoding *CDR1as*. **b** Western blot analysis of p53 and its targets (left); and validation of RNA levels of *CDR1as*, *TP53*, *MDM2*, *CDKN1A* and *PUMA* by RT-qPCR (right) in U87MG cells transfected with different siCDR1as or siNC. **c** Western blot analysis of p53 and its targets in *CDR1as* knocked-down U87MG cells treated with Nutlin3 or DMSO. **d** Luciferase reporter assays for p53 transcription activity in U87MG cells transfected with increasing concentrations of plasmid encoding *CDR1as* (left); and in U87MG cells transfected with different siCDR1as or siNC (right). **e**, **f** Immunoblot of p53 and MDM2 protein and quantification of p53 relative level at the indicated time in U87MG cells transfected with plasmid encoding *CDR1as* or control plasmid (**e**); and in U87MG cells transfected with siCDR1as-1 or siNC (**f**) after CHX treatment to block protein synthesis. **g** Immunoblot of p53 ubiquitination in U87MG cells co-transfected with the plasmids encoding *HA-ubiquitin* (*HA-Ub*), *Myc-MDM2* and *CDR1as* after MG132 treatment (left); and validation of *CDR1as* expression by RT-qPCR (right). **h** Immunoblot of p53 ubiquitination in *CDR1as* knocked-down (or siNC treated) U87MG cells transfected with the plasmids encoding *HA-Ub* and *Myc-MDM2* after MG132 treatment (left); and validation of *CDR1as* expression by RT-qPCR (right). *p < 0.05; **p < 0.01; ***p < 0.001; ****p < 0.0001

As mentioned above, *CDR1as* not only tightly binds to p53 protein, but also significantly correlates with p53 signaling, suggesting *CDR1as* may be capable of regulating p53 activity. To provide further insight into the function of *CDR1as*, we first investigated whether p53 expression and its transcription activity are affected by *CDR1as* in GBM U87MG cells (Fig. 2a-d) and LN229 cells (Additional file 3: Figure S2A, B). The protein level of p53 was increased in a dose-dependent manner after *CDR1as* being over-expressed (Fig. 2a and Additional file 3: Figure S2A), whereas it was significantly decreased after *CDR1as* knockdown (Fig. 2b). The p53 protein level was rescued by Nutlin3, even after *CDR1as* knockdown (Fig. 2c). The mRNA level of *TP53*, however, was not obviously influenced by this circRNA molecule (Fig. 2a, b and Additional file 3: Figure S2A). Therefore, our results demonstrated *CDR1as* promotes the expression of p53 at the protein but not the mRNA level. p53 is known to be a master transcription factor; hence, p53 luciferase reporter assay was performed to confirm whether the transcriptional activity of endogenous p53 is affected by *CDR1as*. Significantly, the transcriptional activity was increased after *CDR1as* over-expression, whereas it was reduced after *CDR1as* knockdown (Fig. 2d and Additional file 3: Figure S2B). Consistent with these findings, the expression levels of p53 targets also increased with *CDR1as* (Fig. 2a and Additional file 3: Figure S2A) and reduced with decrease in *CDR1as* level (Fig. 2b). Moreover, Western blot and IF assays were carried out to determine the sub-cellular distribution of p53 after *CDR1as* knockdown. The results showed that nuclear p53 was down-regulated specifically following *CDR1as* knockdown, suggesting that *CDR1as* may also promote nuclear translocation of p53 in U87MG cells (Additional file 4: Figure S3). Together, our results demonstrated that *CDR1as,* probably via its interaction with p53, up-regulates p53 expression at the protein rather than the mRNA level, thereby promoting activation of p53 signaling.

### *CDR1as* regulates p53 expression independently of its binding to miRNAs

Our studies revealed that *CDR1as* has a potent role in regulating the expression of p53 protein. With multiple docking sites of miRNAs within its sequence, *CDR1as* is primarily believed to function as a sponge to bind different types of miRNAs*,* particularly *miR-7*. Therefore, we predicted that *CDR1as* functions through its ability to bind miRNAs. It is well-known that both AGO2 and Dicer are essential for miRNA biogenesis and mature, thus depletion of either *AGO2* or *Dicer* will lead to reduction of miRNA expression. In U87MG cells, either *AGO2* or *Dicer* knockdown resulted in a decrease in p53 and its target p21 expression, both at the mRNA and protein levels (Additional file 5: Figure S4A-D). Consistently, the transcription activity of p53 was also inhibited by silencing of *AGO2* or *Dicer* (Additional file 5: Figure S4A, C). Enforced expression of *CDR1as*, however, significantly up-regulated the expression levels of p53 and p21, as well as the transcription activity of p53 in U87MG cells where *AGO2* or *Dicer* was knocked down (Additional file 5: Figure S4B, D). Of note, *CDR1as* only rescued p53 expression at the protein level and not at the mRNA level after *AGO2* or *Dicer* silencing (Additional file 5: Figure S4B, D). However, it had little effect on rescuing expression of both mRNA and protein of *AGO2* and *Dicer* in U87MG cells in which *AGO2* or *Dicer* was knocked down (Additional file 5: Figure S4B, D). Moreover, the *miR-7* inhibitor was also found to have no effects on expression of p53 protein whether *CDR1as* was knocked-down or not (Additional file 5: Figure S4E). Contradictory to our initial hypothesis, these results suggested that the function of *CDR1as* in regulating p53 protein is, at least partially, independent of its capacity to bind to miRNAs, particularly *miR-7*.

### *CDR1as* stabilizes p53 protein by suppressing its ubiquitination

When protein synthesis is blocked by CHX, p53 gradually degrades with time. GBM cells were transfected with plasmid encoding *CDR1as* or control plasmid, and the degradation of p53 protein was monitored after CHX treatment. Compared to control, *CDR1as* evidently prevented p53 degradation, thus prolonging its half-life. (Fig. 2e and Additional file 3: Figure S2C). However, when cells were depleted of endogenous *CDR1as* using *CDR1as* siRNA, degradation of p53 protein was increased and therefore its half-life was decreased over time (Fig. 2f). Clearly, these results suggested that *CDR1as* significantly elevates p53 stability, very probably through prohibiting its degradation.

It is well-known that p53 protein is predominantly degraded through the ubiquitin-proteasome pathway [38]. In consequence, we next examined if p53 ubiquitination is affected by *CDR1as*. Co-expression of ubiquitin and MDM2 (the major E3 ubiquitinase of p53) significantly triggered ubiquitination of endogenous p53 protein in GBM cells, while MG132 treatment inhibited p53 degradation (Fig. 2g, h and Additional file 3: Figure S2D). The triggered ubiquitination of p53, however, significantly diminished in cells when *CDR1as* was ectopically expressed (Fig. 2g and Additional file 3: Figure S2D). Meanwhile, p53 ubiquitination was dramatically increased after *CDR1as* was depleted using different siRNAs (Fig. 2h). Clearly, these results demonstrated that *CDR1as* effectively inhibits p53 ubiquitination, therefore preventing p53 protein from degradation through the ubiquitin-proteasome pathway.

### p53 interacts physically with circRNA *CDR1as* via its DBD domain

**Fig. 3:**
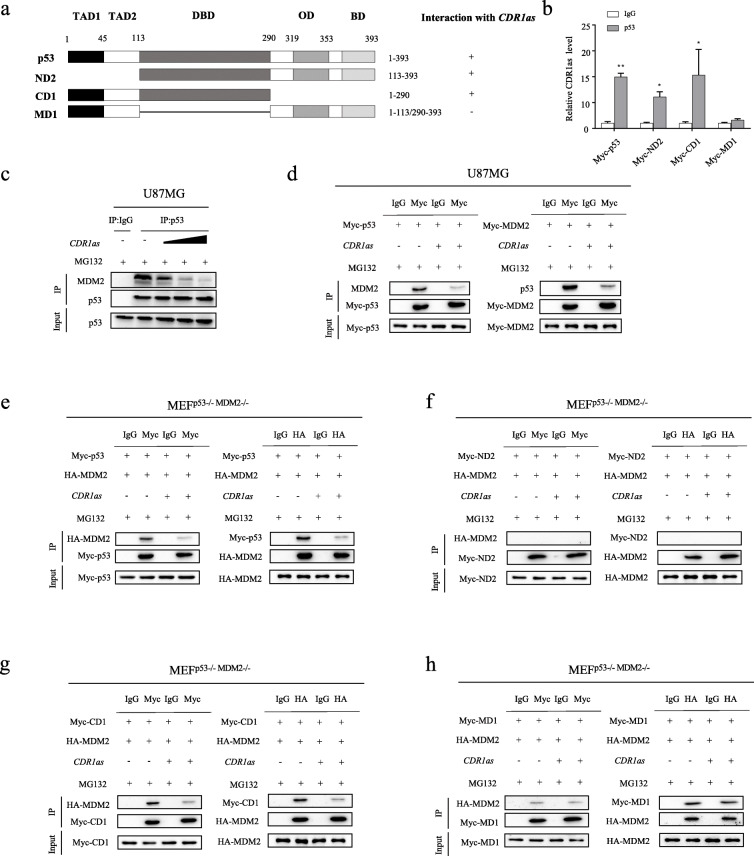
*CDR1as* directly binds with the DBD region of p53 and disrupts the p53/MDM2 complex. **a** A schema showing four constructs containing full-length or different domains of p53. **b** RIP-qPCR analysis of *CDR1as* binding with the indicated p53 constructs. **c** IP analysis of interaction between MDM2 and p53 in U87MG cells transfected with increasing concentrations of plasmid encoding *CDR1as*. **d** IP analysis of interaction between MDM2 and p53 in U87MG cells co-transfected with plasmids encoding *CDR1as*, *Myc-p53* or *Myc-MDM2*. **e-h** IP analysis of interaction between MDM2 and the indicated p53 constructs in MEF DKO (*p53*^*−/−*^*; MDM2*^*−/−*^) cells co-transfected with plasmids encoding *CDR1as*, *HA-MDM2*, and the indicated p53 constructs. *p < 0.05; **p < 0.01

The full-length p53 protein has five functional domains, including two TAD domains, one DBD domain, one OD domain and one BD domain (Fig. 3a). Among them, the DBD and the CTD including OD and BD domains are believed to possess the potential of binding nucleic acids. To analyze the binding capacity of these different domains, four different p53 constructs were generated. As shown in Fig. 3a, they were named as p53-full length, ND2 (deletion of two N-terminal domains), CD1 (deletion of two C-terminal domains) and MD1 (deletion of DBD domains), respectively (Fig. 3a). RIP-qPCR assays were then performed to evaluate their binding capacity to *CDR1as*. Our results showed that only the construct missing the DBD domain lost its capacity to bind *CDR1as*, suggesting that the DBD of p53 is necessary for *CDR1as* binding (Fig. 3b). Although the CTD domain is believed to bind RNA molecules non-specifically, it did not bind the circRNA molecule *CDR1as* in our experimental conditions (Fig. 3b).

### *CDR1as* disrupts the p53/MDM2 complex

The p53 protein is tightly controlled by its negative regulator MDM2, the E3 ubiquitin protein ligase, which promotes p53 ubiquitination and degradation. MDM2 binds tightly to p53 to form the p53/MDM2 complex to facilitate p53 degradation [39, 40]. As demonstrated earlier, *CDR1as* is able to physically bind p53, suggesting that *CDR1as* may impact on the p53/MDM2 complex formation. To test our hypothesis, IP assays were performed. In U87MG cells, the interaction of p53 with MDM2 was significantly inhibited by *CDR1as* in a dose-dependent manner (Fig. 3c). The interaction of the ectopically expressed p53 and MDM2 was hampered by *CDR1as* expression in U87MG cells (Fig. 3d), which was further confirmed in HCT116 cells (Additional file 6: Figure S5A). Therefore, our results suggested that the p53/MDM2 complex can be disrupted by *CDR1as*. MDM2 has been reported to be able to bind RNAs, but our analysis showed that MDM2 does not bind to *CDR1as* directly (data not shown). Thus, our data suggested that *CDR1as* may disrupt the p53/MDM2 complex, which is probably mediated by its interaction with p53 rather than MDM2.

We demonstrated earlier that the DBD region is necessary for the binding of p53 to *CDR1as* (Fig. 3a). Next, we tested if this interaction is involved in restricting the p53/MDM2 complex formation. To this end, plasmids encoding *MDM2* and different *p53* mutant constructs were introduced into MEF DKO (*p53*^−/−^, *MDM2*^−/−^) and the interactions between MDM2 and the distinct p53 constructs were investigated by IP assays. Like full-length p53 (Fig. 3e), the CD1 (Fig. 3g) and MD1 (Fig. 3h) but not the ND2 (Fig. 3f, lacking the N terminal) mutant constructs bound to MDM2 (Fig. 3e-h). However, the interaction between MDM2 and the full-length (Fig. 3e) or the CD1 of p53 (Fig. 3g) was significantly inhibited when *CDR1as* was ectopically expressed. In contrast, *CDR1as* had little effect on the binding of MDM2 to the p53 MD1 mutant that was depleted of the p53 DBD domain (Fig. 3h). In agreement, IP assays from p53^−/−^ HCT116 cells transfected with plasmids encoding full-length, ND2, MD1 and CD1 p53 constructs respectively showed that *CDR1as* can effectively disrupt the binding of endogenous MDM2 to p53 only when DBD domain is intact (Additional file 6: Figure S5B-E). Thus, the p53 DBD domain is required for *CDR1as* to disrupt the p53/MDM2 complex.

Taken together, our results suggested that *CDR1as* is able to disrupt the p53/MDM2 complex through its interaction with the p53 DBD, resulting in an increase in p53 stability through inhibiting its MDM2-mediated ubiquitination and subsequent degradation. Therefore, *CDR1as* depletion may significantly promotes tumorigenesis by p53 inactivation in glioma.

### *CDR1as* suppresses gliomagenesis in vitro and in vivo

We demonstrated that the circRNA *CDR1as* is substantially down-regulated in glioma. More importantly, we discovered that its expression correlates reversely with tumor progression and positively with patient prognosis. These results imply that *CDR1as* may play a potent regulatory role in inhibiting gliomagenesis.

**Fig. 4:**
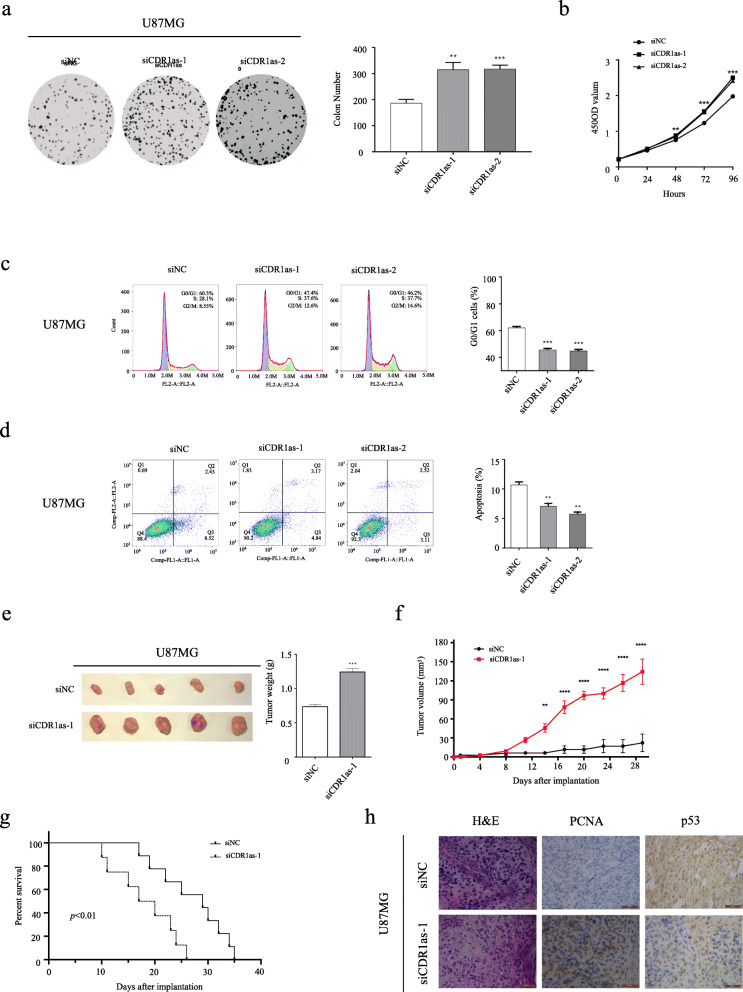
*CDR1as* suppresses gliomagenesis of U87MG cells in vitro and in vivo*.* **a-d** Colony formation assays (**a**), cell proliferation assays (**b**), flow cytometric cell cycle assays (**c**) and flow cytometric apoptosis assays (**d**) for U87MG cells treated with different siCDR1as or siNC. **e** Excised tumors from nude mice xenografted with U87MG cells treated with siCDR1as-1 or siNC. **f** Volume of xenografted tumors derived from U87MG cells treated with siCDR1as-1 or siNC. **g** Kaplan-Meier curves of the overall survival of nude mice xenografted with U87MG cells treated with siCDR1as-1 or siNC. **h** IHC assays for xenografted tumors derived from U87MG cells stained with H&E, PCNA antibody and p53 antibody respectively. *p < 0.05; **p < 0.01; ***p < 0.001; **** *p* < 0.0001

In vitro analyses showed that when *CDR1as* was knocked down in GBM U87MG cells, colony formation (Fig. 4a), cell proliferation (Fig. 4b), and cell division (Fig. 4c) substantially increased, whereas apoptosis (Fig. 4d) decreased. Consistent with this, when *CDR1as* was ectopically expressed in GBM LN229 cells, tumor growth is inhibited (Additional file 7: Figure S6A-D). Therefore, the result demonstrated that *CDR1as* functions as a tumor suppressor and that tumor growth is accelerated with *CDR1as* silencing.

To evaluate the role of *CDR1as* on gliomagenesis in vivo, xenograft mouse models were established by implanting human glioma cells near the axillary fossae of the nude mice. When *CDR1as* was knocked-down in U87MG cells, tumor volume (Fig. 4e, f) was dramatically increased, while overall survival was decreased (Fig. 4g). In xenograft tumors with *CDR1as* knocked-down, p53 expression was significantly down-regulated, whereas cell proliferation was increased (Fig. 4h). When *CDR1as* was ectopically expressed in LN229 cells, however, tumor volume (Additional file 7: Figure S6E, F) was dramatically decreased, and overall survival increased (Additional file 7: Figure S6G). In xenograft tumors with *CDR1as* over-expression, p53 expression was significantly up-regulated, whereas cell proliferation was decreased (Additional file 7: Figure S6H). Taken together, our results showed *CDR1as* has a potent role in suppressing gliomagenesis in vivo.

### *CDR1as* functions in a p53-dependent manner

**Fig. 5:**
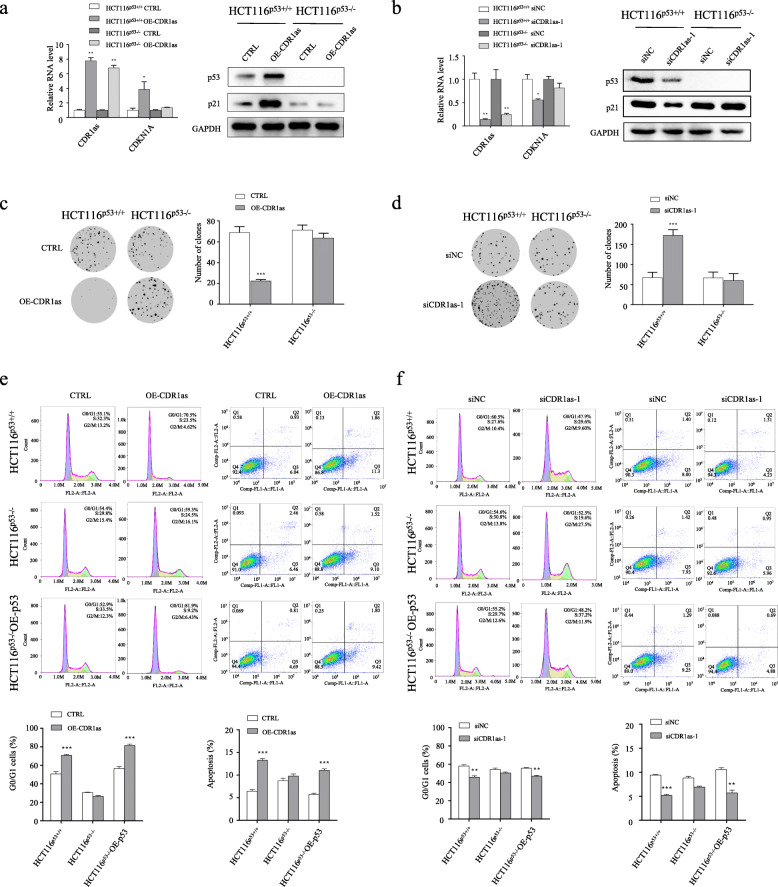
*CDR1as* functions in a p53-dependent manner. **a**, **b** Ectopic expression (**a**), and knock-down (**b**) of *CDR1as* in HCT116 cells in which p53 is intact (HCT116^p53+/+^) or absent (HCT116^p53−/−^). **c**, **d** Colony formation assays for HCT116^p53+/+^ cells and HCT116^p53−/−^ cells in which *CDR1as* is ectopically expressed (**c**) or knocked down (**d**). **e** Flow cytometric cell cycle assays (left) and apoptosis assays (right) for the indicated cells transfected with *CDR1as* expressing plasmid (or control plasmid). **f** Flow cytometric cell cycle assays (left) and apoptosis assays (right) for the indicated cells transfected with siCDR1as-1 (or siNC). *p < 0.05; **p < 0.01; ***p < 0.001

Our work showed *CDR1as* significantly inhibits tumor growth and that p53 activity is affected by *CDR1as* expression (Fig. 2). These results suggested that it is possible for *CDR1as* to function in a p53-dependent manner. Therefore, the influence of *CDR1as* over-expression (Fig. 5a) or knock-down (Fig. 5b) in cells in which p53 is functional (p53^+/+^) or absent (p53^−/−^) were compared. In HCT116 ^p53+/+^ cells and not HCT116 ^p53−/−^ cells, enforced expression of *CDR1as* significantly inhibited colony formation and cell proliferation, whereas knockdown of *CDR1as* promoted colony formation and cell proliferation (Fig. 5c, d). Next, we further evaluated the impact of *CDR1as* on cell cycle and apoptosis in cells where p53 is wild-type or deleted. The results shown in Fig. 5e and Fig. 5f revealed that *CDR1as* promoted G0/G1 arrest and apoptosis in HCT116 ^p53+/+^ cells, but had little effect on HCT116 ^p53−/−^ cells. After p53 was reintroduced into p53 null cells, *CDR1as*, regained its ability to cause cell cycle arrest and apoptosis (Fig. 5e, f). Next, the tumor suppressive role of *CDR1as* was investigated further in the p53 mutant GBM T98G (Additional file 8: Figure S7A-D) and U251 (Additional file 8: Figure S7E-H) cells. The results showed that enforced expression or knockdown of *CDR1as* had little effect on colony formation (Additional file 8: Figure S7A, E) and cell proliferation (Additional file 8: Figure S7B, F) in these cells in which p53 is inactivated due to mutation. In addition, enforced expression or knockdown of *CDR1as* did not confer to significant changes of cell cycle (Additional file 8: Figure S7C, G) and apoptosis (Additional file 8: Figure S7D, H) in these cells too. Taken together, our results demonstrated that *CDR1as* suppresses tumorigenesis of glioma in a p53-dependent manner.

### *CDR1as* protects p53 function against DNA damage

**Fig. 6:**
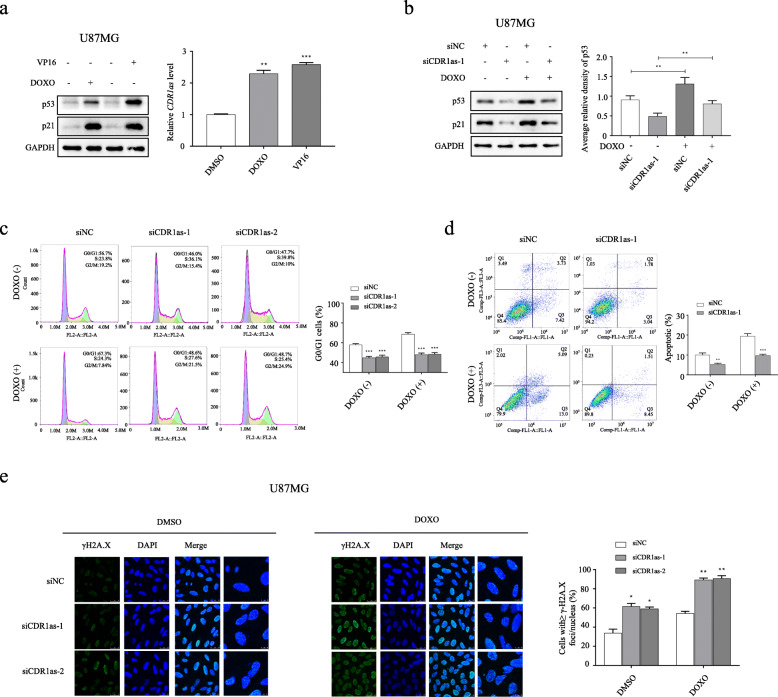
*CDR1as* serves as a protective machinery to preserve p53 function against DNA damage in U87MG cells. **a** Western blot analysis of p53 and p21 expression (left); and RT-qPCR analysis of *CDR1as* expression (right) in U87MG cells after 48 h treatment with DOXO, VP16 or DMSO (as control). **b** Western blot analysis of p53 and p21 expression (left); and densitometric analysis of p53 expression normalized to GAPDH (right) in U87MG cells transfected with siCDR1as-1 or siNC after 48 h treatment of DOXO or DMSO. **c** Flow cytometric analysis of cell cycle in U87MG cells transfected with different siCDR1as or siNC after 48 h treatment of DOXO or DMSO. **d** Flow cytometric analysis of apoptosis in U87MG cells transfected with siCDR1as-1 or siNC after 48 h treatment of DOXO or DMSO. **e** IF analysis of γH2A.X (DNA damage marker) in U87MG cells transfected with different siCDR1as or siNC after 48 h treatment of DOXO or DMSO (left); quantification of number of γH2A.X positive cells with equal or more than 10 γH2A.X foci/nucleus (right). *p < 0.05; ** *p* < 0.01; ***p < 0.001

The expression level of *CDR1as* is dramatically elevated in different cell types following treatment with the DNA damaging agents, DOXO and VP16 (Fig. 6a and Additional file 9: Figure S8A). *CDR1as,* as well as p53 signaling, is simultaneously activated upon DNA damage, implying that *CDR1as* is a DNA-damage induced lncRNA molecule. Indeed, being closely co-localized with p53 in nucleus, *CDR1as* promoted p53 accumulation in cells treated with DOXO, indicating it may be critical for preserving p53 function in response to DNA damage (Fig. 6b and Additional file 9: Figure S8B). Flow cytometric assays showed that *CDR1as* silencing bypassed the DNA damage-induced G0/G1 arrest (Fig. 6c) and apoptosis (Fig. 6d) in cells treated with DOXO, whereas enforced expression of *CDR1as* promotes cell cycle arrest (Additional file 9: Figure S8C) and apoptosis (Additional file 9: Figure S8D) in cells upon DNA damage. To confirm further the impact of *CDR1as* on DNA damage, IF assays on phosphorylated histone H2AX (γH2A.X) foci which reflect the degree of DNA damage were carried out. Our results showed that *CDR1as* knockdown significantly induced the formation of γH2A.X foci in U87MG cells (Fig. 6e). Enforced expression of *CDR1as* in LN229 cells, however, causes a dramatic decrease in γH2A.X foci (Additional file 9: Figure S8E). Taken together, the data suggest that *CDR1as* is not only a sensor of DNA damage but also a mediator of DNA damage response. Upon DNA damage, *CDR1as* is induced to protect and to stabilize p53 and to reduce the accumulation of damage DNA.

## Discussion

It is well established that p53 is a master suppressor of tumorigenesis and loss of p53 functions has been found to occur in almost all tumors by either mutation or inactivation [3]. Glioma, particular GBM, usually harbors a structurally intact *TP53* gene [5], indicating the augmented proliferation and resistance to cytotoxic treatment in GBM is attributable to the loss of p53 function by inactivation [6]. p53 is one of the most studied molecules of all time, but our understanding of its regulatory and signaling network remains incomplete. Ample evidence supports the notion that lncRNAs play a key role in tumorigenesis [12, 13]. Recently, the emergence of circRNAs has further reshaped and widened our insights into eukaryotic transcripts and their roles [14, 16, 18, 19]. CircRNAs, however, are among the least well characterized non-coding transcripts, and our knowledge of their function in cancer development is very limited. Particularly, whether or how the master tumor suppressor p53 is regulated by circRNAs is yet to be explored.

DBD and CTD are two distinct nucleic acid-binding domains of p53 [23]. It is believed that p53-RNA interaction which may modulate p53 activity is mediated by the CTD rather than the core DBD [41–43]. Hitherto, whether p53 interacts with lncRNAs, particularly with circular RNAs, and the relevant physiological significance remains almost unknown. Through RNA immunoprecipitation analysis, we found multiple lncRNAs bind to the p53 protein. Among them, the circRNA *CDR1as* interacts with p53 and is significantly correlated to p53 signaling. Being particularly abundant in brain, *CDR1as* is essential for normal development and function of brain [28]. Meanwhile, *CDR1as* is also found to be implicated in tumorigenesis [26, 27]. However, the published work shows contradictory results relating to its impact on tumorigenesis [25, 26], suggesting that the underlying mechanisms require more carefully dissection. *CDR1as* has previously been established as a miRNA sponge [26–28]. Our present results reveal that *CDR1as* is strongly bound by p53, which suggests that circRNAs, as exemplified by *CDR1as*, may represent a new layer of control of the tumorigenic process, functioning via their interaction with proteins.

p53 is a key suppressor of tumorigenesis [3, 4]. Our initial assay proves that the p53 level is significantly affected by *CDR1as*. The protein level of p53 is substantially down-regulated by *CDR1as* inactivation, while the mRNA level does not change much. Further analysis shows that *CDR1as* positively regulates the stability of p53 protein through limiting its ubiquitination and degradation. Therefore, our results suggest that *CDR1as* may function as a tumor suppressor through restricting p53 degradation. *CDR1as* is one of a few of circRNA molecules whose roles and the underlying mechanisms of action have begun to be characterized, and have been found to primarily function as a miRNA sponge [25–28]. A few of initial findings on its role in tumorigenesis is, however, mutual contradictory [25, 26]. In this study, we verify that *CDR1as* is substantially down-regulated in glioma, the most common and yet incurable tumor in CNS. *CDR1as* dramatically inhibits tumor growth in a p53-dependent way. Rather than being a miRNA sponge, *CDR1as* is able to bind physically to p53 protein, thus preventing its ubiquitination by MDM2 and subsequent degradation. Evidently, we demonstrate that *CDR1as* may restrict tumorigenesis of glioma through stabilizing the p53 protein.

It is known that MDM2, an E3 ubiquitin ligase, is a key regulator of p53 stability through the 26S proteasome-degradation pathway. Being a crucial oncogenic molecule, MDM2 is frequently over-expressed in numerous cancers [44, 45]. MDM2 complexes with p53 to modulate the level and thereby, the transcriptional activity of p53. The stability of p53/MDM2 complex is modulated by many factors that bind either p53 or MDM2. The interaction of the p53/MDM2 complex with p300 [8] promotes p53 degradation, whereas its binding to Rb [46], c-Abl [10] or ARF [47] stabilizes p53. Both p53 [41, 43, 48] and MDM2 [49, 50] can bind RNAs, indicating the interactions between p53/MDM2 with RNAs may influence p53 stability. *CDR1as* binds p53 instead of MDM2, suggesting that the interaction of *CDR1as* with the p53/MDM2 complex is most likely to be through p53. This is further verified by the fact that *CDR1as* is commonly co-localized with p53 protein. More importantly, *CDR1as* inhibits tumor growth in a p53-dependent manner. Upon DNA damage, *CDR1as* is significantly up-regulated, which results in accumulation of p53 protein to promote cell cycle arrest. Our work demonstrates that *CDR1as* complexes with p53 protein*,* which may sense signaling of DNA damage and serve as a protective machinery to preserve p53 function.

The primary docking sites of p53 with MDM2 is located in the N-terminal region of p53. Crystallographic analysis shows that the N-terminus of p53 forms an amphipathic α-helix, of which three hydrophobic and aromatic residues are inserted into MDM2 cleft, thus forming a complex [51]. Although it is well established that MDM2 interacts with the N-terminal domain of p53 to induce its degradation, the p53/MDM2 interaction can also be affected by factors binding to regions away from p53 N-terminal domain [52]. Binding-induced allosteric changes of p53 at regions distal from its N-terminus may play important roles in its function and regulation. It has been reported that DNA binding through its DBD region promotes conformation change of p53 protein, thus protecting it from interacting with partners [53]. Therefore, we speculate that DBD binding to circRNA CDR1as can also provoke p53 conformational changes, leading to its N-terminus dissociation from MDM2, and ultimately limiting p53 degradation. Notably, the DBD domain of p53 also forms a key interface for MDM2 binding, which has been demonstrated to be essential for its ubiquitination and degradation [54]. In consequence, *CDR1as* binding can potentially restrict the interaction of p53 with MDM2, resulting in the arrest of p53 ubiquitination and degradation.

Collectively, our results show that *CDR1as* plays a vital role as a tumor suppressor of glioma tumorigenesis. *CDR1as* is expressed abundantly in brain, but at low levels in most of other normal tissues [28]. Compared to adjacent normal brain, *CDR1as* is greatly down-regulated in glioma [20]. It restricts cell proliferation by inducing cycle arrest and apoptosis. Therefore, *CDR1as* possesses the inhibitory function of tumorigenesis, which is further verified by the experiments of tumor growth in vivo. Notably, the present findings are at odds with those of others who reported that *CDR1as* functions as an oncogene [26, 27]. It is well known that gene functions are context dependent, so many genes can possess dual functions as tumor suppressors or oncogenes. *CDR1as* has previously been regarded as a potent sponge for miRNAs, particularity *miR-7* ^27–28^. Our results, for the first time, discover that it can bind protein and it may function by destabilizing the p53/MDM2 complex independently of its binding with miRNAs. With further study in the future, more aspects of its function will be disclosed.

Tumorigenesis is most frequently driven by loss of wild-type p53 function, either due to its mutations or inactivation [3]. p53 stability is closely controlled by the formation of p53/MDM2 complex. We first discover that circRNA *CDR1as* directly interacts with this complex, and their interaction may disrupt of the binding of p53 with MDM2, thus preventing formation of the p53/MDM2 complex. In this manner, *CDR1as* stabilizes p53 protein through decreasing ubiquitination by MDM2. In glioma, down-regulated *CDR1as* may promote tumorigenesis due to p53 inactivation. For decades, targeting the p53/MDM2 complex has been one of top attractive strategies of cancer treatment [45, 55]. Our discovery might pave a way for a strategy to overcome one of the greatest challenges of health care.

## Supplementary information

**Additional file 1 Table S1**. Multivariate Cox regression analysis of prognosis in glioma. *p < 0.05; **p < 0.01. **Table S2**. The list of qPCR primers. **Table S3**. The list of siRNA sequence.

**Additional file 2 Figure S1**. *CDR1as* expression in different types of human tissue and cancer. **A.** Heatmap of expression of p53-binding lncRNAs in human tissues (up); *CDR1as* expression in human tissues (low). **B.** Heatmap of expression of p53-binding lncRNAs in 31 types of cancer (up); *CDR1as* expression in 31 types of cancer (low). **C.** Kaplan-Meier curves of the overall survival of patients for *CDR1as* with different glioma grades in the CGGA cohort. **D.** AUC (Area Under Curve) plotted for different durations of survival for p53-binding lncRNAs in the CGGA cohort (up); Cox univariate regression for survival for p53-binding lncRNAs in the CGGA cohort (low). **E.** RNA FISH assays of *CDR1as* expression in glioma (*n* = 87) and normal brain tissues (*n* = 3). *p < 0.05; **p < 0.01; ***p < 0.001; ****p < 0.0001.

**Additional file 3 Figure S2**. *CDR1as* up-regulates expression of p53 protein by inhibiting its ubiquitination in LN229 cells. **A.** Western bolt analysis of p53 and its targets (left); and validation of RNA levels of *CDR1as*, *TP53*, *MDM2*, *CDKN1A* and *PUMA* by RT-qPCR (right) in LN229 cells transfected with increasing concentrations of plasmid encoding *CDR1as*. **B.** Luciferase reporter assays for p53 transcription activity in LN229 cells transfected with increasing concentrations of plasmid encoding *CDR1as*. **C.** Immunoblot of p53 protein (left) and quantification of its relative level (right) at the indicated time in LN229 cells transfected with plasmid encoding *CDR1as* or control plasmid with CHX treatment to block protein synthesis. **D.** Immunoblot of p53 ubiquitination in LN229 cells co-transfected with the plasmids encoding *HA-Ub*, *Myc-MDM2* and *CDR1as* with MG132 treatment to inhibit proteasomal degradation. *p < 0.05; **p < 0.01; ***p < 0.001.

**Additional file 4 Figure S3**. *CDR1as* regulates sub-cellular distribution of p53 in U87MG cells. U87MG cells were transfected with different siCDR1as or siNC. After 48 h, cells were treated with MG132 for 4 h. Subsequently, cell fractionation assays (**A**) were performed for cytoplasmic and nuclear fraction of p53. Fractionation efficiency was validated by Western blot using antibodies specific to marker proteins of each fraction: GAPDH for cytoplasm and Histone 3 (H3) for nucleus. IF assays (**B**) were performed for sub-cellular localization of p53.

**Additional file 5 Figure S4**. *CDR1as* stabilizes p53 protein independently on its binding with miRNAs. **A.** RT-qPCR assays for RNAs of *AGO2*, *CDR1as*, *TP53* and *CDKN1A* expression (up); Western blot assays for proteins of AGO2, p53 and p21 (middle); and luciferase reporter assays for p53 transcription activity (low) in U87MG cells transfected with different siAGO2 or siNC. **B.** RT-qPCR assays for RNAs of *AGO2*, *CDR1as*, *TP53* and *CDKN1A* expression (up); Western blot assays for proteins of AGO2, p53 and p21 (middle); and luciferase reporter assays for p53 transcription activity (low) in *AGO2* knocked down U87MG cells transfected with plasmid encoding *CDR1as* or control plasmid. **C.** RT-qPCR assays for RNAs of *Dicer*, *CDR1as*, *TP53* and *CDKN1A* expression (up); Western blot assays for proteins of Dicer, p53 and p21 (middle); and luciferase reporter assays for p53 transcription activity (low) in U87MG cells transfected with different siDicer or siNC. **D.** RT-qPCR assays for RNAs of *Dicer*, *CDR1as*, *TP53* and *CDKN1A* expression (up); Western blot assays for proteins of Dicer, p53 and p21 (middle); and luciferase reporter assays for p53 transcription activity (low) in *Dicer* knocked down U87MG cells transfected with plasmid encoding *CDR1as* or control plasmid. **E.** Western blot analysis of p53 and its targets in U87MG cells transfected with siCDR1as or not (NC) 48 h after treatment with the *miR-7* inhibitor (General Biosystems, 25 nM); RT-qPCR analysis of *miR-7*, *CDR1as*, *TP53*, *MDM2* and *CDKN1A* in U87MG cells 48 h after treatment with the *miR-7* inhibitor. ns, no significance; *p < 0.05; **p < 0.01; ***p < 0.001.

**Additional file 6 Figure S5**. *CDR1as* prevents the binding between p53 and MDM2 in HCT116 cells. **A.** IP analysis of binding between MDM2 and p53 in HCT116^p53+/+^ cells co-transfected with plasmids encoding *CDR1as*, and *Myc-p53* or *Myc-MDM2* after MG132 treatment. **B-E.** IP analysis of MDM2 binding with full-length p53 (**B**), ND2 (**C**), CD1 (**D**) and MD1 (**E**) respectively in HCT116^p53−/−^ cells co-transfected with the indicated constructs after MG132 treatment.

**Additional file 7 Figure S6**. *CDR1as* suppresses gliomagenesis of LN229 cells in vitro and in vivo*.* **A-D.** Colony formation assays (**A**), cell proliferation assays (**B**), flow cytometric cell cycle assays (**C**), and flow cytometric apoptosis assays (**D**) for LN229 cells transfected with *CDR1as* expressing plasmid or control plasmid. **E.** Excised tumors from nude mice xenografted with LN229 cells transfected with *CDR1as* expressing plasmid or control plasmid. **F.** Volume of xenografted tumors derived from LN229 cells transfected with *CDR1as* expressing plasmid or control plasmid. **G.** Kaplan-Meier curves of the overall survival of nude mice xenografted with LN229 cells transfected with *CDR1as* expressing plasmid or control plasmid. **H.** IHC assays for xenografted tumors derived from the indicated LN229 cells stained with H&E, PCNA antibody and p53 antibody respectively. *p < 0.05; **p < 0.01; ***p < 0.001; ****p < 0.0001.

**Additional file 8 Figure S7**. *CDR1as* has little effects on growth of p53-mutant GBM cells T98G and U251. **A-B.** Colony formation assays (**A**), and cell proliferation assays (**B**) for p53 mutant T98G cells in which *CDR1as* expression was manipulated. **C-D.** Flow cytometric cell cycle assays (**C**) and apoptosis assays (**D**) for p53 mutant T98G cells in which *CDR1as* expression was manipulated after 48 h treatment with DOXO or DMSO. **E-F.** Colony formation assays (**E**), and cell proliferation assays (**F**) for p53 mutant U251 cells in which *CDR1as* expression was manipulated. **G-H.** Flow cytometric cell cycle assays (**G**) and apoptosis assays (**H**) for p53 mutant U251 cells in which *CDR1as* expression was manipulated after 48 h treatment with DOXO or DMSO. *p < 0.05, **p < 0.01, ***p < 0.001, ****p < 0.0001.

**Additional file 9 Figure S8**. *CDR1as* serves as a protective machinery to preserve p53 function against DNA damage in LN229 cells. **A.** Western blot analysis of p53 and p21 expression (left), and RT-qPCR analysis of *CDR1as* expression (right) in LN229 cells after 48 h treatment of DOXO, VP16 or DMSO. **B.** Immunoblot of p53 and p21 (left), and densitometric analysis of p53 expression normalized to GAPDH (right) in LN229 cells transfected with plasmid encoding *CDR1as* or control plasmid after 48 h treatment of DOXO or DMSO. **C.** Flow cytometric analysis of cell cycle in LN229 cells transfected with plasmid encoding *CDR1as* or control plasmid after 48 h treatment of DOXO or DMSO. **D.** Flow cytometric analysis of apoptosis in LN229 cells transfected with plasmid encoding *CDR1as* or control plasmid after 48 h treatment of DOXO or DMSO. **E.** IF analysis of γH2A.X in LN229 cells transfected with plasmid encoding *CDR1as* or control plasmid after 48 h treatment of DOXO or DMSO (left); quantification of number of γH2A.X positive cells with equal or more than 10 γH2A.X foci/nucleus (right). *p < 0.05; **p < 0.01.

**Additional file 10 Figure S9**. Ubiquitination of p53 in U87MG cells without MG132 treatment. **A.** Immunoblot of p53 ubiquitination in U87MG cells co-transfected with the plasmids encoding *HA-Ub*, *Myc-MDM2* and *CDR1as* without MG132 treatment. **B.** Immunoblot of p53 ubiquitination in *CDR1as* knocked-down (or siNC treated) U87MG cells transfected with the plasmids encoding *HA-Ub* and *Myc-MDM2* without MG132 treatment.

## Data Availability

All data generated or analyzed during this study are included in this published article and its Additional files.

## References

[1] Ostrom QT, Cioffi G, Gittleman H, Patil N, Waite K, Kruchko C, Barnholtz-Sloan JS (2019). CBTRUS statistical report: primary brain and other central nervous system tumors diagnosed in the United States in 2012–2016. Neuro Oncol.

[2] Khasraw M, Lassman AB (2010). Advances in the treatment of malignant gliomas. Curr Oncol Rep.

[3] Kastenhuber ER, Lowe SW (2017). Putting p53 in context. Cell.

[4] Vogelstein B, Lane D, Levine AJ (2000). Surfing the p53 network. Nature.

[5] Research CGA (2008). N., comprehensive genomic characterization defines human glioblastoma genes and core pathways. Nature.

[6] Mehta S, Huillard E, Kesari S, Maire CL, Golebiowski D, Harrington EP, Alberta JA, Kane MF, Theisen M, Ligon KL, Rowitch DH, Stiles CD (2011). The central nervous system-restricted transcription factor Olig2 opposes p53 responses to genotoxic damage in neural progenitors and malignant glioma. Cancer Cell.

[7] Brennan CW, Verhaak RG, McKenna A, Campos B, Noushmehr H, Salama SR, Zheng S, Chakravarty D, Sanborn JZ, Berman SH, Beroukhim R, Bernard B, Wu CJ, Genovese G, Shmulevich I, Barnholtz-Sloan J, Zou L, Vegesna R, Shukla SA, Ciriello G, Yung WK, Zhang W, Sougnez C, Mikkelsen T, Aldape K, Bigner DD, Van Meir EG, Prados M, Sloan A, Black KL, Eschbacher J, Finocchiaro G, Friedman W, Andrews DW, Guha A, Iacocca M, O'Neill BP, Foltz G, Myers J, Weisenberger DJ, Penny R, Kucherlapati R, Perou CM, Hayes DN, Gibbs R, Marra M, Mills GB, Lander E, Spellman P, Wilson R, Sander C, Weinstein J, Meyerson M, Gabriel S, Laird PW, Haussler D, Getz G, Chin L, Network TR (2013). The somatic genomic landscape of glioblastoma. Cell.

[8] Grossman SR, Perez M, Kung AL, Joseph M, Mansur C, Xiao ZX, Kumar S, Howley PM, Livingston DM (1998). p300/MDM2 complexes participate in MDM2-mediated p53 degradation. Mol Cell.

[9] Hsieh JK, Chan FS, O'Connor DJ, Mittnacht S, Zhong S, Lu X (1999). RB regulates the stability and the apoptotic function of p53 via MDM2. Mol Cell.

[10] Sionov RV, Moallem E, Berger M, Kazaz A, Gerlitz O, Ben-Neriah Y, Oren M, Haupt Y (1999). C-Abl neutralizes the inhibitory effect of Mdm2 on p53. J Biol Chem.

[11] Zhang Y, Xiong Y (1999). Mutations in human ARF exon 2 disrupt its nucleolar localization and impair its ability to block nuclear export of MDM2 and p53. Mol Cell.

[12] Anastasiadou E, Jacob LS, Slack FJ (2018). Non-coding RNA networks in cancer. Nat Rev Cancer.

[13] Esposito R, Bosch N, Lanzos A, Polidori T, Pulido-Quetglas C, Johnson R (2019). Hacking the Cancer genome: profiling therapeutically actionable long non-coding RNAs using CRISPR-Cas9 screening. Cancer Cell.

[14] Jeck WR, Sorrentino JA, Wang K, Slevin MK, Burd CE, Liu J, Marzluff WF, Sharpless NE (2013). Circular RNAs are abundant, conserved, and associated with ALU repeats. RNA.

[15] Kristensen LS, Andersen MS, Stagsted LVW, Ebbesen KK, Hansen TB, Kjems J (2019). The biogenesis, biology and characterization of circular RNAs. Nat Rev Genet.

[16] Salzman J, Chen RE, Olsen MN, Wang PL, Brown PO (2013). Cell-type specific features of circular RNA expression. PLoS Genet.

[17] Nicolet BP, Engels S, Aglialoro F, van den Akker E, von Lindern M, Wolkers MC (2018). Circular RNA expression in human hematopoietic cells is widespread and cell-type specific. Nucleic Acids Res.

[18] Barrett SP, Salzman J (2016). Circular RNAs: analysis, expression and potential functions. Development.

[19] Salzman J (2016). Circular RNA expression: its potential regulation and function. Trends Genet.

[20] Song X, Zhang N, Han P, Moon BS, Lai RK, Wang K, Lu W (2016). Circular RNA profile in gliomas revealed by identification tool UROBORUS. Nucleic Acids Res.

[21] Vo JN, Cieslik M, Zhang Y, Shukla S, Xiao L, Zhang Y, Wu YM, Dhanasekaran SM, Engelke CG, Cao X, Robinson DR, Nesvizhskii AI, Chinnaiyan AM (2019). The landscape of circular RNA in Cancer. Cell.

[22] Chen N, Zhao G, Yan X, Lv Z, Yin H, Zhang S, Song W, Li X, Li L, Du Z, Jia L, Zhou L, Li W, Hoffman AR, Hu JF, Cui J (2018). A novel FLI1 exonic circular RNA promotes metastasis in breast cancer by coordinately regulating TET1 and DNMT1. Genome Biol.

[23] Liu Y, Kulesz-Martin MF (2006). Sliding into home: facilitated p53 search for targets by the basic DNA binding domain. Cell Death Differ.

[24] Du WW, Fang L, Yang W, Wu N, Awan FM, Yang Z, Yang BB (2017). Induction of tumor apoptosis through a circular RNA enhancing Foxo3 activity. Cell Death Differ.

[25] Li P, Yang X, Yuan W, Yang C, Zhang X, Han J, Wang J, Deng X, Yang H, Li P, Tao J, Lu Q, Gu M (2018). CircRNA-*CDR1as* exerts anti-oncogenic functions in bladder Cancer by sponging MicroRNA-135a. Cell Physiol Biochem.

[26] Sang M, Meng L, Sang Y, Liu S, Ding P, Ju Y, Liu F, Gu L, Lian Y, Li J, Wu Y, Zhang X, Shan B (2018). Circular RNA ciRS-7 accelerates ESCC progression through acting as a miR-876-5p sponge to enhance MAGE-A family expression. Cancer Lett.

[27] Memczak S, Jens M, Elefsinioti A, Torti F, Krueger J, Rybak A, Maier L, Mackowiak SD, Gregersen LH, Munschauer M, Loewer A, Ziebold U, Landthaler M, Kocks C, le Noble F, Rajewsky N (2013). Circular RNAs are a large class of animal RNAs with regulatory potency. Nature.

[28] Piwecka M, Glazar P, Hernandez-Miranda LR, Memczak S, Wolf SA, Rybak-Wolf A, Filipchyk A, Klironomos F, Cerda Jara CA, Fenske P, Trimbuch T, Zywitza V, Plass M, Schreyer L, Ayoub S, Kocks C, Kuhn R, Rosenmund C, Birchmeier C, Rajewsky N. Loss of a mammalian circular RNA locus causes miRNA deregulation and affects brain function. Science. 2017;357(6357):eaam8526. 10.1126/science.aam8526 28798046

[29] Chu C, Zhang QC, da Rocha ST, Flynn RA, Bharadwaj M, Calabrese JM, Magnuson T, Heard E, Chang HY (2015). Systematic discovery of Xist RNA binding proteins. Cell.

[30] Chu C, Qu K, Zhong FL, Artandi SE, Chang HY (2011). Genomic maps of long noncoding RNA occupancy reveal principles of RNA-chromatin interactions. Mol Cell.

[31] Cui Y, Hu D, Markillie LM, Chrisler WB, Gaffrey MJ, Ansong C, Sussel L, Orr G (2018). Fluctuation localization imaging-based fluorescence in situ hybridization (fliFISH) for accurate detection and counting of RNA copies in single cells. Nucleic Acids Res.

[32] Dobin A, Davis CA, Schlesinger F, Drenkow J, Zaleski C, Jha S, Batut P, Chaisson M, Gingeras TR (2013). STAR: ultrafast universal RNA-seq aligner. Bioinformatics.

[33] Goldman M, Craft B, Hastie M, Repečka K, McDade F, Kamath A, Banerjee A, Luo Y, Rogers D, Brooks A. N, Zhu J, Haussler D. Visualizing and interpreting cancer genomics data via the Xena platform. Nat Biotechnol. 2020;38(6):675–8. 10.1038/s41587-020-0546-8 PMC7386072 32444850

[34] Tang Z, Li C, Kang B, Gao G, Li C, Zhang Z (2017). GEPIA: a web server for cancer and normal gene expression profiling and interactive analyses. Nucleic Acids Res.

[35] Mello SS, Attardi LD (2018). Deciphering p53 signaling in tumor suppression. Curr Opin Cell Biol.

[36] Bao ZS, Chen HM, Yang MY, Zhang CB, Yu K, Ye WL, Hu BQ, Yan W, Zhang W, Akers J, Ramakrishnan V, Li J, Carter B, Liu YW, Hu HM, Wang Z, Li MY, Yao K, Qiu XG, Kang CS, You YP, Fan XL, Song WS, Li RQ, Su XD, Chen CC, Jiang T (2014). RNA-seq of 272 gliomas revealed a novel, recurrent PTPRZ1-MET fusion transcript in secondary glioblastomas. Genome Res.

[37] Suzuki H, Zuo Y, Wang J, Zhang MQ, Malhotra A, Mayeda A (2006). Characterization of RNase R-digested cellular RNA source that consists of lariat and circular RNAs from pre-mRNA splicing. Nucleic Acids Res.

[38] Katz C, Low-Calle AM, Choe JH, Laptenko O, Tong D, Joseph-Chowdhury JN, Garofalo F, Zhu Y, Friedler A, Prives C (2018). Wild-type and cancer-related p53 proteins are preferentially degraded by MDM2 as dimers rather than tetramers. Genes Dev.

[39] Aberg E, Saccoccia F, Grabherr M, Ore WYJ, Jemth P, Hultqvist G (2017). Evolution of the p53-MDM2 pathway. BMC Evol Biol.

[40] Lane DP, Verma C (2012). Mdm2 in evolution. Genes Cancer.

[41] Yoshida Y, Izumi H, Torigoe T, Ishiguchi H, Yoshida T, Itoh H, Kohno K (2004). Binding of RNA to p53 regulates its oligomerization and DNA-binding activity. Oncogene.

[42] Riley KJ, Ramirez-Alvarado M, Maher LJ (2007). RNA-p53 interactions in vitro. Biochemistry.

[43] Riley KJ, Maher LJ (2007). p53 RNA interactions: new clues in an old mystery. RNA.

[44] Momand J, Jung D, Wilczynski S, Niland J (1998). The MDM2 gene amplification database. Nucleic Acids Res.

[45] Wang S, Zhao Y, Aguilar A, Bernard D, Yang C. Y. Targeting the MDM2-p53 Protein-Protein Interaction for New Cancer Therapy: Progress and Challenges. Cold Spring Harb Perspect Med. 2017;7(5):a026245. 10.1101/cshperspect.a026245 PMC5411684 28270530

[46] Cui J, Wang L, Ren X, Zhang Y, Zhang H (2019). LRPPRC: a multifunctional protein involved in energy metabolism and human disease. Front Physiol.

[47] Rolland SG, Motori E, Memar N, Hench J, Frank S, Winklhofer KF, Conradt B (2013). Impaired complex IV activity in response to loss of LRPPRC function can be compensated by mitochondrial hyperfusion. Proc Natl Acad Sci U S A.

[48] Vikhanskaya F, Lee MK, Mazzoletti M, Broggini M, Sabapathy K (2007). Cancer-derived p53 mutants suppress p53-target gene expression--potential mechanism for gain of function of mutant p53. Nucleic Acids Res.

[49] Naski N, Gajjar M, Bourougaa K, Malbert-Colas L, Fahraeus R, Candeias MM (2009). The p53 mRNA-Mdm2 interaction. Cell Cycle.

[50] Gajjar M, Candeias MM, Malbert-Colas L, Mazars A, Fujita J, Olivares-Illana V, Fahraeus R (2012). The p53 mRNA-Mdm2 interaction controls Mdm2 nuclear trafficking and is required for p53 activation following DNA damage. Cancer Cell.

[51] Kussie PH, Gorina S, Marechal V, Elenbaas B, Moreau J, Levine AJ, Pavletich NP (1996). Structure of the MDM2 oncoprotein bound to the p53 tumor suppressor transactivation domain. Science.

[52] Lambrughi M, De Gioia L, Gervasio FL, Lindorff-Larsen K, Nussinov R, Urani C, Bruschi M, Papaleo E (2016). DNA-binding protects p53 from interactions with cofactors involved in transcription-independent functions. Nucleic Acids Res.

[53] Kovachev PS, Banerjee D, Rangel LP, Eriksson J, Pedrote MM, Martins-Dinis M, Edwards K, Cordeiro Y, Silva JL, Sanyal S (2017). Distinct modulatory role of RNA in the aggregation of the tumor suppressor protein p53 core domain. J Biol Chem.

[54] Wallace M, Worrall E, Pettersson S, Hupp TR, Ball KL (2006). Dual-site regulation of MDM2 E3-ubiquitin ligase activity. Mol Cell.

[55] Khoo KH, Verma CS, Lane DP (2014). Drugging the p53 pathway: understanding the route to clinical efficacy. Nat Rev Drug Discov.

