# Supplementary material for: Circular RNA CDR1as disrupts the p53/MDM2 complex to inhibit Gliomagenesis
Source: Mol Cancer. 2020 Sep 7;19:138. doi: 10.1186/s12943-020-01253-y (PMC7487905; doi:10.1186/s12943-020-01253-y)
Supplement: Supplementary file 1 — Additional file 1 Table S1. Multivariate Cox regression analysis of prognosis in glioma. *p < 0.05; **p < 0.01. Table S2. The list of qPCR primers. Table S3. The list of siRNA sequence. [file 12943_2020_1253_MOESM1_ESM.zip › TableS1.pdf]

Table S1

Factors	TCGA RNA-seq set					CGGA RNA-seq set				
		Univariate		Multivariate			Univariate		Multivariate	
	sample	p value	HR	p value	HR	sample	p value	HR	p value	HR
Age										
Increaseing years	618	<0.001	1.066	<0.001	1.0626	301	<0.001	1.0405	<0.01	1.0219
Gender										
Male vs Female	618	0.0787	1.265	0.749964	1.0478	301	0.3298	1.1912	0.749964	1.1775
1p19q codeletion										
non-codel vs codel	671	<0.001	4.515	<0.001	3.4318	298	<0.001	9.2908	<0.001	6.9482
MGMT promoter status										
Unmethylated vs methylated	642	<0.001	3.2	<0.001	1.8705					
Radiation										
Yes vs. No						289	<0.01	0.4999	<0.01	0.5497
CDR1_AS										
Increasing expression	732	<0.001	0.8274	<0.05	0.9495	301	<0.001	0.7127	<0.001	0.8346
