# Supplementary material for: Circular RNA CDR1as disrupts the p53/MDM2 complex to inhibit Gliomagenesis
Source: Mol Cancer. 2020 Sep 7;19:138. doi: 10.1186/s12943-020-01253-y (PMC7487905; doi:10.1186/s12943-020-01253-y)
Supplement: Supplementary file 1 — Additional file 1 Table S1. Multivariate Cox regression analysis of prognosis in glioma. *p < 0.05; **p < 0.01. Table S2. The list of qPCR primers. Table S3. The list of siRNA sequence. [file 12943_2020_1253_MOESM1_ESM.zip › TableS2.pdf]

Supplemental Table S3. All primers used in this study

(1) Primer sequences used in RT-PCR and qRT-PCR analysis

Gene		Sequence（5’-3’）
CDR1as	Forward	GTGTCTGCAATATCCAGGGTT
	Reverse	ACTGGAAGACTTGGAGCTTCTTG
TP53	Forward	GCCCACTTCACCGTACTAACCA
	Reverse	GAAACTACCAACCCACCGACCA
CDKN1A	Forward	GTTCCGCACAGGAGCAAAGT
	Reverse	ACGGCGCAACTGCTCAC
MDM2	Forward	CTTTGCAGTAAAATATGCCCTT
	Reverse	GCAAACCTTATTCGGCTCT
PUMA	Forward	TCCTGTGCTCTGCCCGTGAC
	Reverse	CCTCCCTGACTCCCCGTCT
ACTB	Forward	ATGTGGCCGAGGACTTTGATT
	Reverse	AGTGGGGTGGCTTTTAGGATG
U6	Forward	GCTTCGGGAGCACATATACTAAAAT
	Reverse	CGCTTCACGAATTTGCGTGTCAT
GAPDH	Forward	CCAAGGAGTAAGACCCCTGG
	Reverse	TGGTTGAGCACAGGGTACTT
MALAT1	Forward	CCCCTGGGCTTCTCTTAACA
	Reverse	TAGATCAAAAGGCACGGGGT
Dicer	Forward	GAAGAATCAGCCTCGCAAC
	Reverse	TGTGGGCAAATCAAAACGAAC
AGO2	Forward	GGCCGACACAGATATTCCA
	Reverse	TAAAATTTAAACCACCCCGCAGA
RP11-58H15.4	Forward	TGATGTTCCGTCTGCGTCACC
	Reverse	CTCCACCAGTTTATTGCGACGTA
RP11-93B14.5	Forward	GCTCTTTCCTCTCGGCCACA
	Reverse	TAACTAGGGCTGCACCGTCT
RP1-1J6.2	Forward	CCTTGTTATCATGCCGGACT
	Reverse	CTTATTTCACTATCCTCGTGCTC
RP11-520M5.5	Forward	ACACATACCGCCTTTACTCC
	Reverse	TGGTTATCTCACTGTCGGCAA
RP11-456N14.2	Forward	CACGGATGTAGACTGATTCCT
	Reverse	CATTTTACCGTAAGTGATGTCC
RP11-507C10.5	Forward	CAACTTACTGGTTTCGGCAT
	Reverse	CAAGGTTGGCTGATTCCAT
RP11-112L6.5	Forward	CTACTGTGTCCCTGGTGCCTA
	Reverse	AAGCCCTTTTGCAGAACACTC
CACTIN-AS1	Forward	ACGATCTTGAAAGCGATGTCC
	Reverse	CGCACTACGACTTTGACAACCC
BAIAP2-AS1	Forward	CACGCCTGTAATCACAACACT
	Reverse	TCTCGGCTCACTGCAACCTCT
AP006621.8	Forward	CGCTCGGCAGGTCTGAACCC
	Reverse	CGCCCAGCTCCTCCGTCCT
RP11-800A3.7	Forward	TGCTCGCTCCTGGCACCCTT
	Reverse	TCGCCTCAGTCTGCAAGTCCT
RP11-284F21.10	Forward	AAATGGGCACTCCGACCAC
	Reverse	CGCCGAGATTTCCGACCTTG
RP11-157P1.4	Forward	GCAAATTTTCACCTTCGCATT
	Reverse	TATGCCCCACAAACATTCCG
RP11-575L7.8	Forward	GGGCATTCAATCAGAAGTTGTC
	Reverse	CATATATCTCCGTTGCCCAT
RP11-57H14.2	Forward	AAAACCCCACCAGCCCGAG
	Reverse	CCTTCAAAGACGAGGGCGAAC
RP11-324O2.3	Forward	TCATCTCCGAATTGGCTTG
	Reverse	CTTGAACATTTTCCGACCAC

LL22NC03-86G7.1	Forward	AAAACATCTGTGCGATCTCCC
	Reverse	TTGTGCGTTCACCCTTTATCCTT
RP13-1032I1.7	Forward	CCAGCACTTTAGGAGCCCGAG
	Reverse	CCTCCTGGGTTCAAGCGAT
RP5-1142A6.9	Forward	TGGCCTTGAGCAGTACCTCC
	Reverse	GAATTCGTGCGTCAGACCACT
RP5-1142A6.2	Forward	AGACCCGTCACAGCCTACCAC
	Reverse	TTTCCCATCAGCACTCGGGGCTT
RP11-299J3.8	Forward	CCTCCACTTTAATGAGACCGAT
	Reverse	TGCACCCACTGTCCAACCA
AC068580.6	Forward	ACGATGACCAACCCCACGAC
	Reverse	TCTTTGCATCCTCCACGGCTC
CTC-518P12.6	Forward	AGGCAATACTGAATACCAGAG
	Reverse	TTTGTTTACACCGATGTCCCT
RP11-580I16.2	Forward	AAGCCCAGTAGTGAAAGCTA
	Reverse	GTTTATTACCAGGCATTCGT
RP11-823E8.3	Forward	CGCAAGAAACACCACAAGCA
	Reverse	CAAATTGGCACATCTTAGCTCT
RP11-452I5.2	Forward	GCCGAGAGAGGACCACAGACCAA
	Reverse	TGTCTCCCGCCTCTGTGACCA
CTD-3193O13.12	Forward	CTTTAGACGGCTTTCGCTGA
	Reverse	TTACCTGCATCTACACGCTCT
RP5-940J5.6	Forward	TCCTTAGAATCGTATGGCACT
	Reverse	TTCTTGCAGCTCCCCGTAG
CTC-444N24.11	Forward	AATATTTCAAGTGGCTAACCC
	Reverse	TTCTAATCATGCAACCCGACA
circPLXNA1	Forward	GATCCTCAAGGCGCACCTCT
	Reverse	CGTGACGCGCGTGCATCCAC

(2) Primer sequences of *CDR1as* used in RNA-Pulldown

CDR1as Probe	*CDR1as* _p1	GTTGTTGGAAGACCTTGACA
	CDR1as _p2	GAAGACGTGGATTTTCCTGG
	CDR1as _p3	GATTGACTGGAAGACCTGGA
	CDR1as _p4	GAAGACCTGGATTTTTTCCG
	CDR1as _p5	CCGGAAGACTTGGATTGTTG
	CDR1as _p6	GGAAGACATGGATAGTCTGG
	CDR1as _p7	CTGGAAGACATGGCTTTGTT
	CDR1as _p8	GTTGGAAGACGTGGATTTGC
	CDR1as _p9	CCTTTGTTGGAAGACATACC
	CDR1as _p10	GGAAGACGTAGCTTAGTTGG
	CDR1as _p11	GCTGGAAGACACAGAGATGC
	CDR1as _p12	ATTGACGACACTGGAAGACC
