# Supplementary material for: Circular RNA CDR1as disrupts the p53/MDM2 complex to inhibit Gliomagenesis
Source: Mol Cancer. 2020 Sep 7;19:138. doi: 10.1186/s12943-020-01253-y (PMC7487905; doi:10.1186/s12943-020-01253-y)
Supplement: Supplementary file 1 — Additional file 1 Table S1. Multivariate Cox regression analysis of prognosis in glioma. *p < 0.05; **p < 0.01. Table S2. The list of qPCR primers. Table S3. The list of siRNA sequence. [file 12943_2020_1253_MOESM1_ESM.zip › TableS3.pdf]

Supplemental Table S3. All RNAi Sequences used in this study

Gene	Sequence（5’-3’）
Scramble siSCR NC	CAGATTCGCGAATGTACGTTT
CDR1as siRNA1	GCACCTGTGTCAAGGTCTTTT
CDR1as siRNA2	GGTCTTCCAGCGACTTCAATT
Dicer siRNA1	TTTGTTGCGAGGCTGATTC
Dicer siRNA2	TCTATTAGCACCTTGAUGT
AGO2 siRNA1	CAGACUCCCGUGUGUCCUATT
AGO2 siRNA2	CGGACAAUCAGACCUCCACTT
CTRL inhibitor	UCUACUCUUUCUAGGAGGUUGUGA
miR-7 inhibitor	AACAACAAAAUCACUAGUCUUCCA
