# Supplementary material for: Circular RNA CDR1as disrupts the p53/MDM2 complex to inhibit Gliomagenesis
Source: Mol Cancer. 2020 Sep 7;19:138. doi: 10.1186/s12943-020-01253-y (PMC7487905; doi:10.1186/s12943-020-01253-y)
Supplement: Supplementary file 2 — Additional file 2 Figure S1. CDR1as expression in different types of human tissue and cancer. A. Heatmap of expression of p53-binding lncRNAs in human tissues (up); CDR1as expression in human tissues (low). B. Heatmap of expression of p53-binding lncRNAs in 31 types of cancer (up); CDR1as expression in 31 types of cancer (low). C. Kaplan-Meier curves of the overall survival of patients for CDR1as with different glioma grades in the CGGA cohort. D. AUC (Area Under Curve) plotted for different durations of survival for p53-binding lncRNAs in the CGGA cohort (up); Cox univariate regression for survival for p53-binding lncRNAs in the CGGA cohort (low). E. RNA FISH assays of CDR1as expression in glioma (n = 87) and normal brain tissues (n = 3). *p < 0.05; **p < 0.01; ***p < 0.001; ****p < 0.0001. [file 12943_2020_1253_MOESM2_ESM.pdf]

A

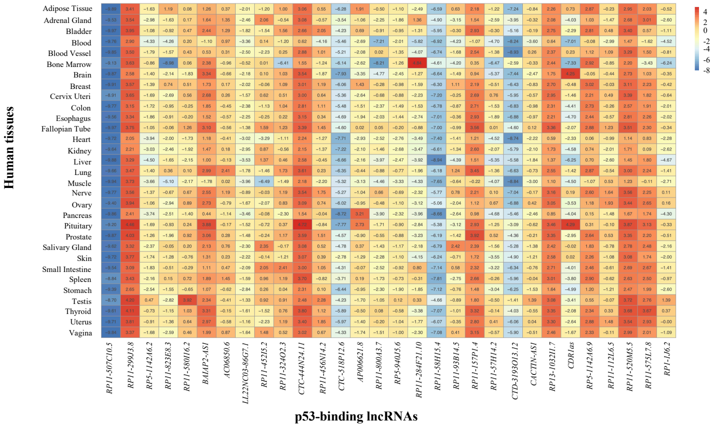

Expression of p53-binding lncRNAs in human tissues (GTEx dataset)

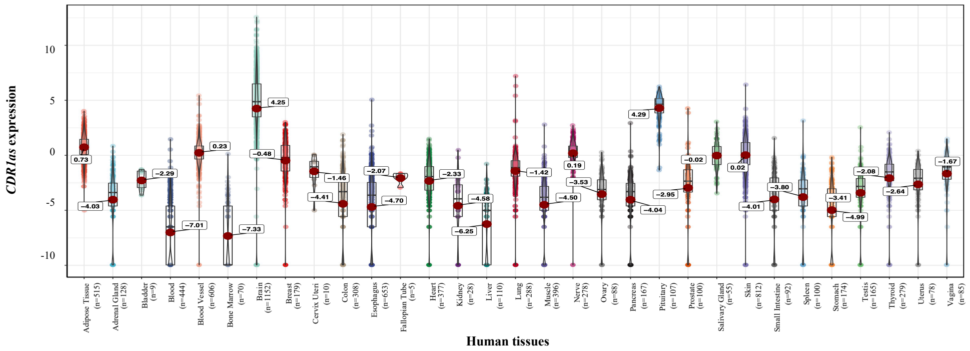

Expression of *CDR1as* in human tissues (GTEx dataset)

Additional file 2: Figure S1

B

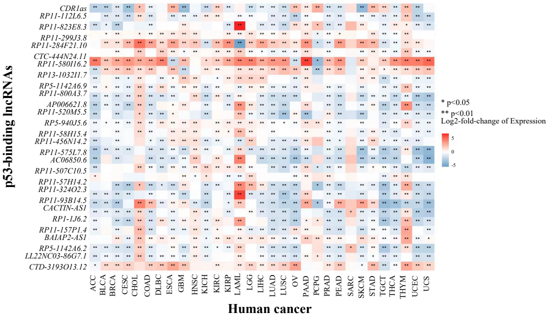

Expression of p53-binding lncRNAs in Pan-Cancer

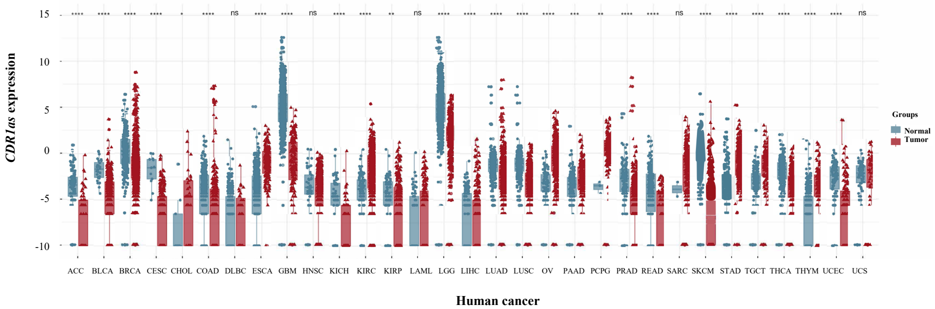

Expression of *CDR1as* in Pan-Cancer (GTEx dataset)

C

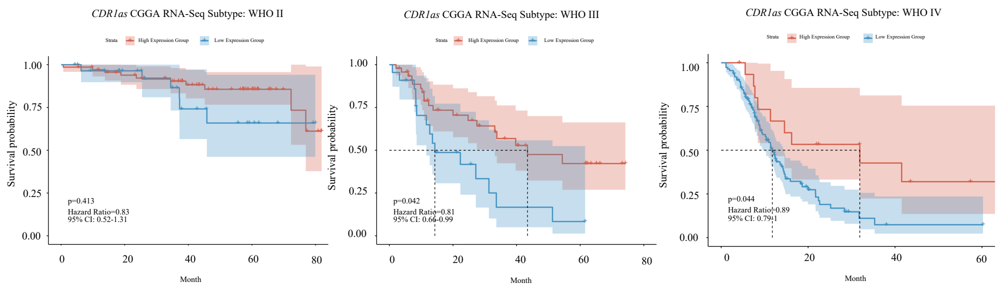

Kaplan-Meier survival curves for *CDR1as* with different glioma grades in CGGA cohort

D

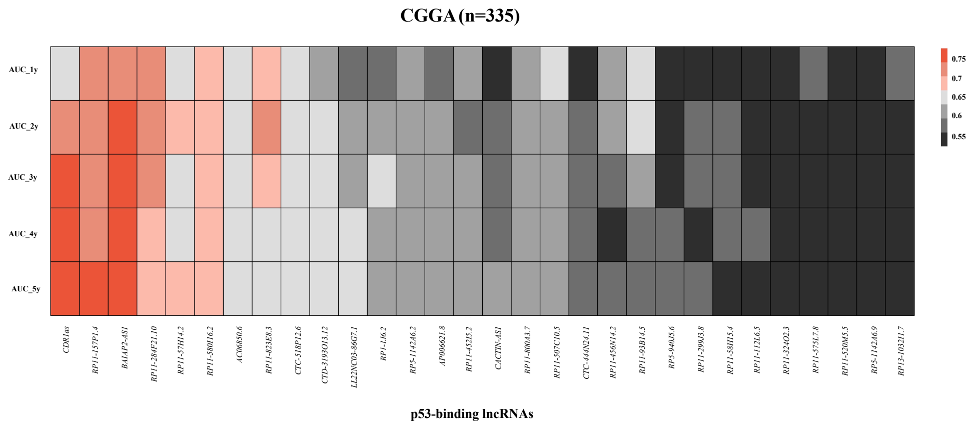

AUC for different durations of survival for p53-binding lncRNAs in CGGA cohort

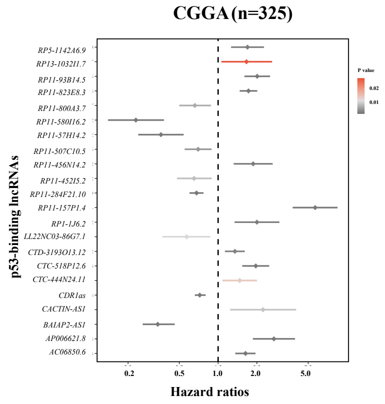

Univariate Cox regression analysis

Additional file 2: Figure S1

E

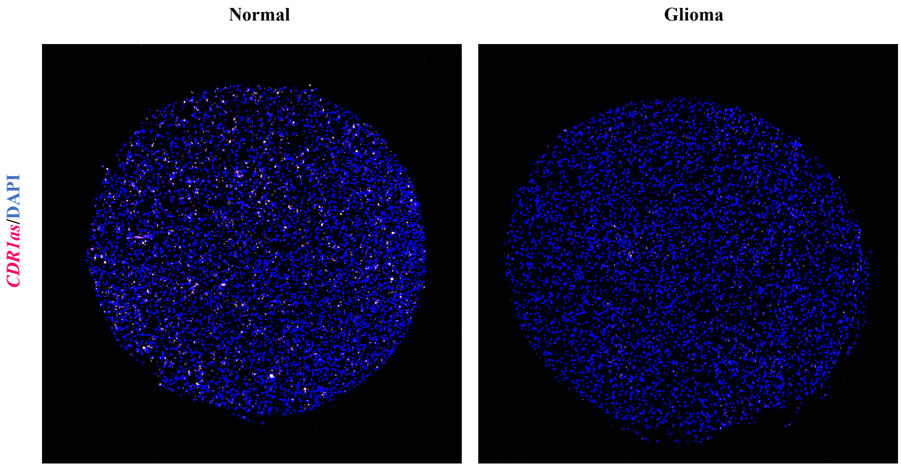

RNA FISH assays for *CDR1as* expression in glioma and normal brain tissues
