# Supplementary figures and images for: Circular RNA CDR1as disrupts the p53/MDM2 complex to inhibit Gliomagenesis
Source: Mol Cancer. 2020 Sep 7;19:138. doi: 10.1186/s12943-020-01253-y (PMC7487905; doi:10.1186/s12943-020-01253-y)

A

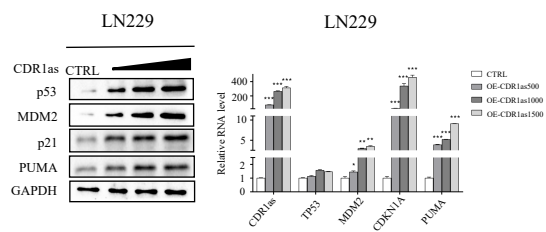

B

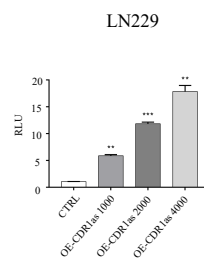

C

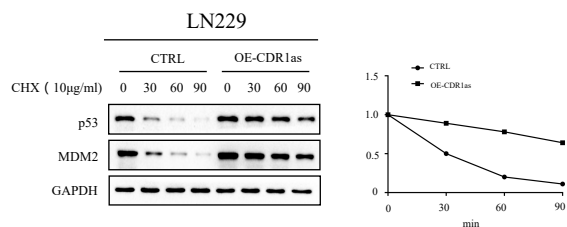

D

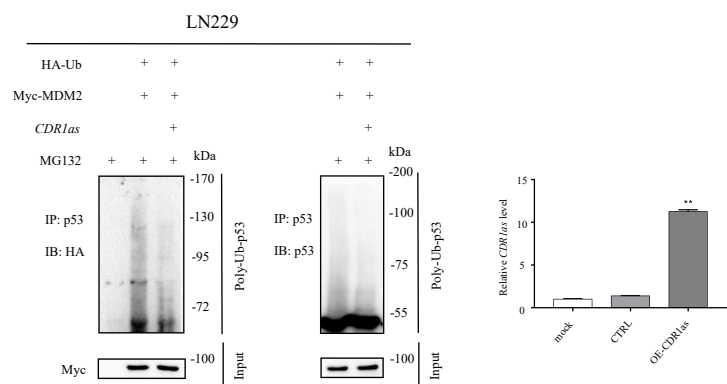

Supplement: Supplementary file 3 — Additional file 3 Figure S2. CDR1as up-regulates expression of p53 protein by inhibiting its ubiquitination in LN229 cells. A. Western bolt analysis of p53 and its targets (left); and validation of RNA levels of CDR1as, TP53, MDM2, CDKN1A and PUMA by RT-qPCR (right) in LN229 cells transfected with increasing concentrations of plasmid encoding CDR1as. B. Luciferase reporter assays for p53 transcription activity in LN229 cells transfected with increasing concentrations of plasmid encoding CDR1as. C. Immunoblot of p53 protein (left) and quantification of its relative level (right) at the indicated time in LN229 cells transfected with plasmid encoding CDR1as or control plasmid with CHX treatment to block protein synthesis. D. Immunoblot of p53 ubiquitination in LN229 cells co-transfected with the plasmids encoding HA-Ub, Myc-MDM2 and CDR1as with MG132 treatment to inhibit proteasomal degradation. *p < 0.05; **p < 0.01; ***p < 0.001. [file 12943_2020_1253_MOESM3_ESM.pdf]

A

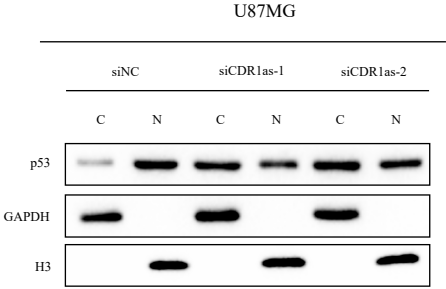

B

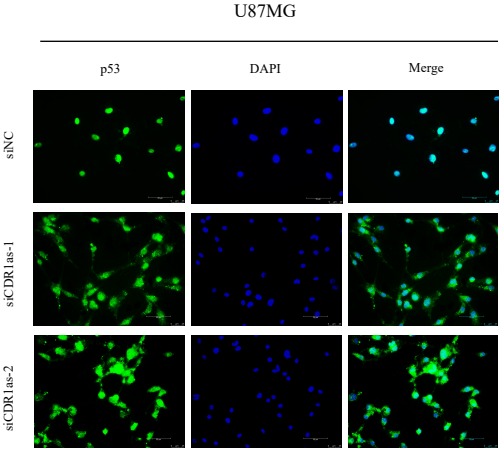

Supplement: Supplementary file 4 — Additional file 4 Figure S3. CDR1as regulates sub-cellular distribution of p53 in U87MG cells. U87MG cells were transfected with different siCDR1as or siNC. After 48 h, cells were treated with MG132 for 4 h. Subsequently, cell fractionation assays (A) were performed for cytoplasmic and nuclear fraction of p53. Fractionation efficiency was validated by Western blot using antibodies specific to marker proteins of each fraction: GAPDH for cytoplasm and Histone 3 (H3) for nucleus. IF assays (B) were performed for sub-cellular localization of p53. [file 12943_2020_1253_MOESM4_ESM.pdf]

A

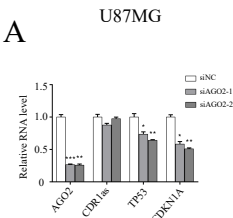

B

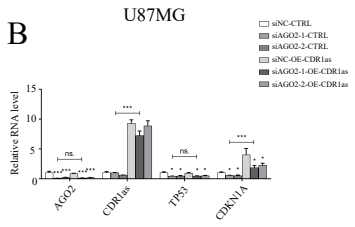

C

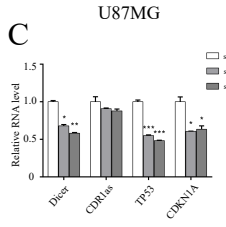

D

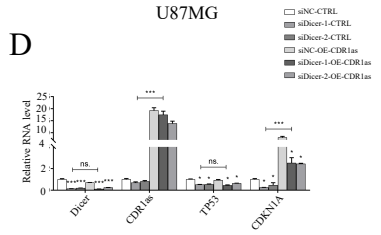

E

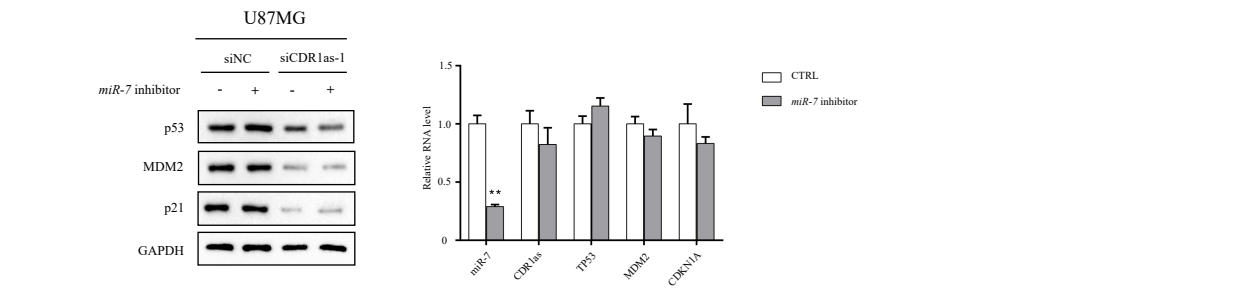

Supplement: Supplementary file 5 — Additional file 5 Figure S4. CDR1as stabilizes p53 protein independently on its binding with miRNAs. A. RT-qPCR assays for RNAs of AGO2, CDR1as, TP53 and CDKN1A expression (up); Western blot assays for proteins of AGO2, p53 and p21 (middle); and luciferase reporter assays for p53 transcription activity (low) in U87MG cells transfected with different siAGO2 or siNC. B. RT-qPCR assays for RNAs of AGO2, CDR1as, TP53 and CDKN1A expression (up); Western blot assays for proteins of AGO2, p53 and p21 (middle); and luciferase reporter assays for p53 transcription activity (low) in AGO2 knocked down U87MG cells transfected with plasmid encoding CDR1as or control plasmid. C. RT-qPCR assays for RNAs of Dicer, CDR1as, TP53 and CDKN1A expression (up); Western blot assays for proteins of Dicer, p53 and p21 (middle); and luciferase reporter assays for p53 transcription activity (low) in U87MG cells transfected with different siDicer or siNC. D. RT-qPCR assays for RNAs of Dicer, CDR1as, TP53 and CDKN1A expression (up); Western blot assays for proteins of Dicer, p53 and p21 (middle); and luciferase reporter assays for p53 transcription activity (low) in Dicer knocked down U87MG cells transfected with plasmid encoding CDR1as or control plasmid. E. Western blot analysis of p53 and its targets in U87MG cells transfected with siCDR1as or not (NC) 48 h after treatment with the miR-7 inhibitor (General Biosystems, 25 nM); RT-qPCR analysis of miR-7, CDR1as, TP53, MDM2 and CDKN1A in U87MG cells 48 h after treatment with the miR-7 inhibitor. ns, no significance; *p < 0.05; **p < 0.01; ***p < 0.001. [file 12943_2020_1253_MOESM5_ESM.pdf]

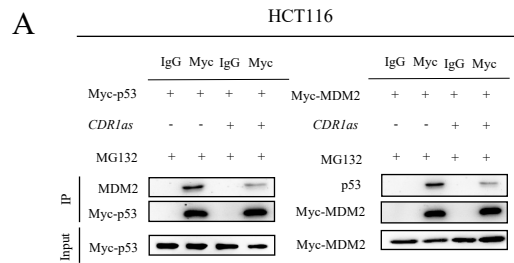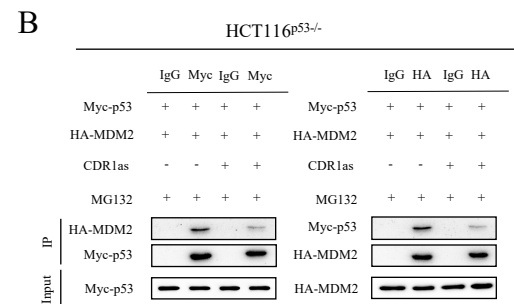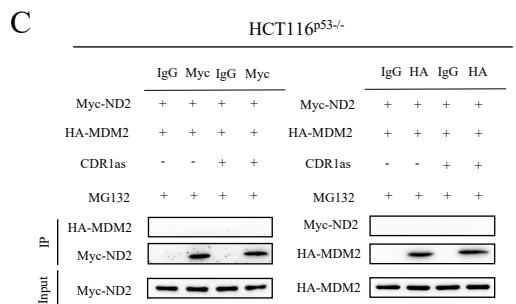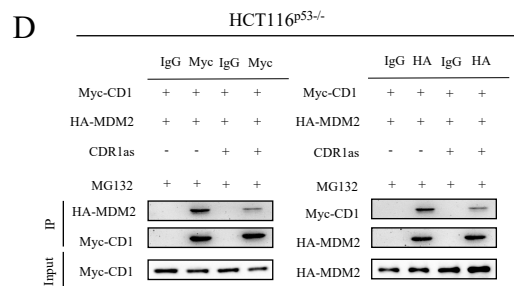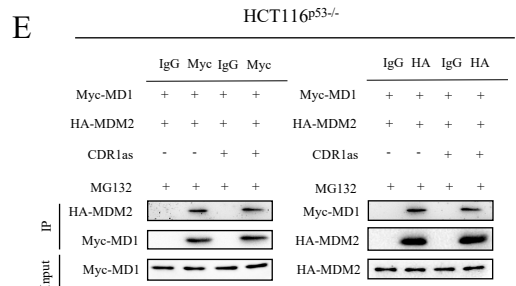

Supplement: Supplementary file 6 — Additional file 6 Figure S5. CDR1as prevents the binding between p53 and MDM2 in HCT116 cells. A. IP analysis of binding between MDM2 and p53 in HCT116p53+/+ cells co-transfected with plasmids encoding CDR1as, and Myc-p53 or Myc-MDM2 after MG132 treatment. B-E. IP analysis of MDM2 binding with full-length p53 (B), ND2 (C), CD1 (D) and MD1 (E) respectively in HCT116p53−/− cells co-transfected with the indicated constructs after MG132 treatment. [file 12943_2020_1253_MOESM6_ESM.pdf]

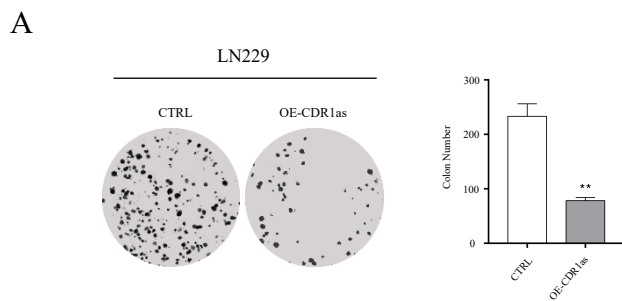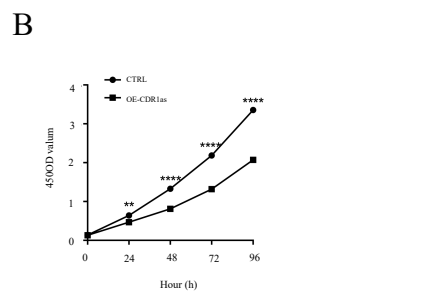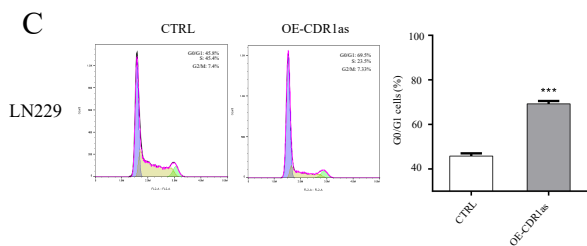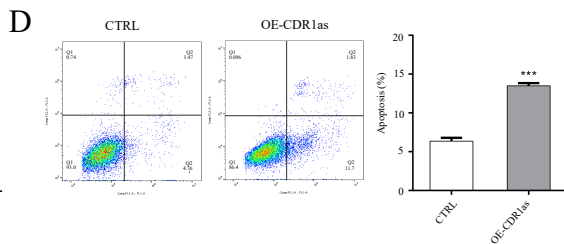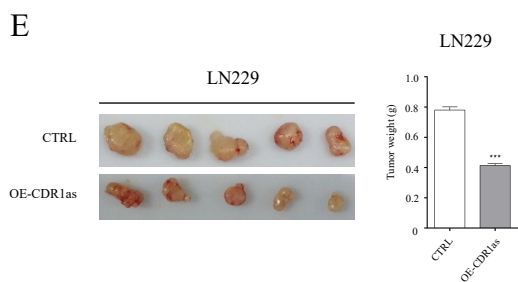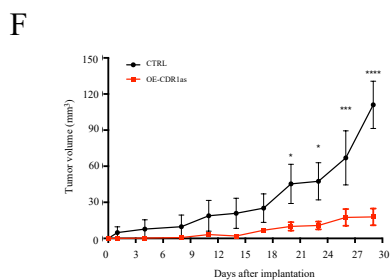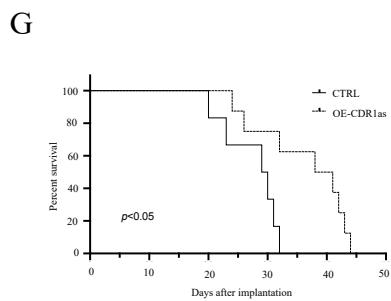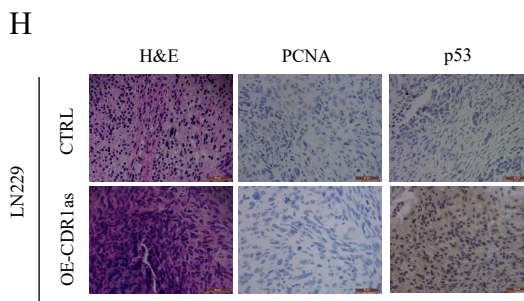

Supplement: Supplementary file 7 — Additional file 7 Figure S6. CDR1as suppresses gliomagenesis of LN229 cells in vitro and in vivo. A-D. Colony formation assays (A), cell proliferation assays (B), flow cytometric cell cycle assays (C), and flow cytometric apoptosis assays (D) for LN229 cells transfected with CDR1as expressing plasmid or control plasmid. E. Excised tumors from nude mice xenografted with LN229 cells transfected with CDR1as expressing plasmid or control plasmid. F. Volume of xenografted tumors derived from LN229 cells transfected with CDR1as expressing plasmid or control plasmid. G. Kaplan-Meier curves of the overall survival of nude mice xenografted with LN229 cells transfected with CDR1as expressing plasmid or control plasmid. H. IHC assays for xenografted tumors derived from the indicated LN229 cells stained with H&E, PCNA antibody and p53 antibody respectively. *p < 0.05; **p < 0.01; ***p < 0.001; ****p < 0.0001. [file 12943_2020_1253_MOESM7_ESM.pdf]

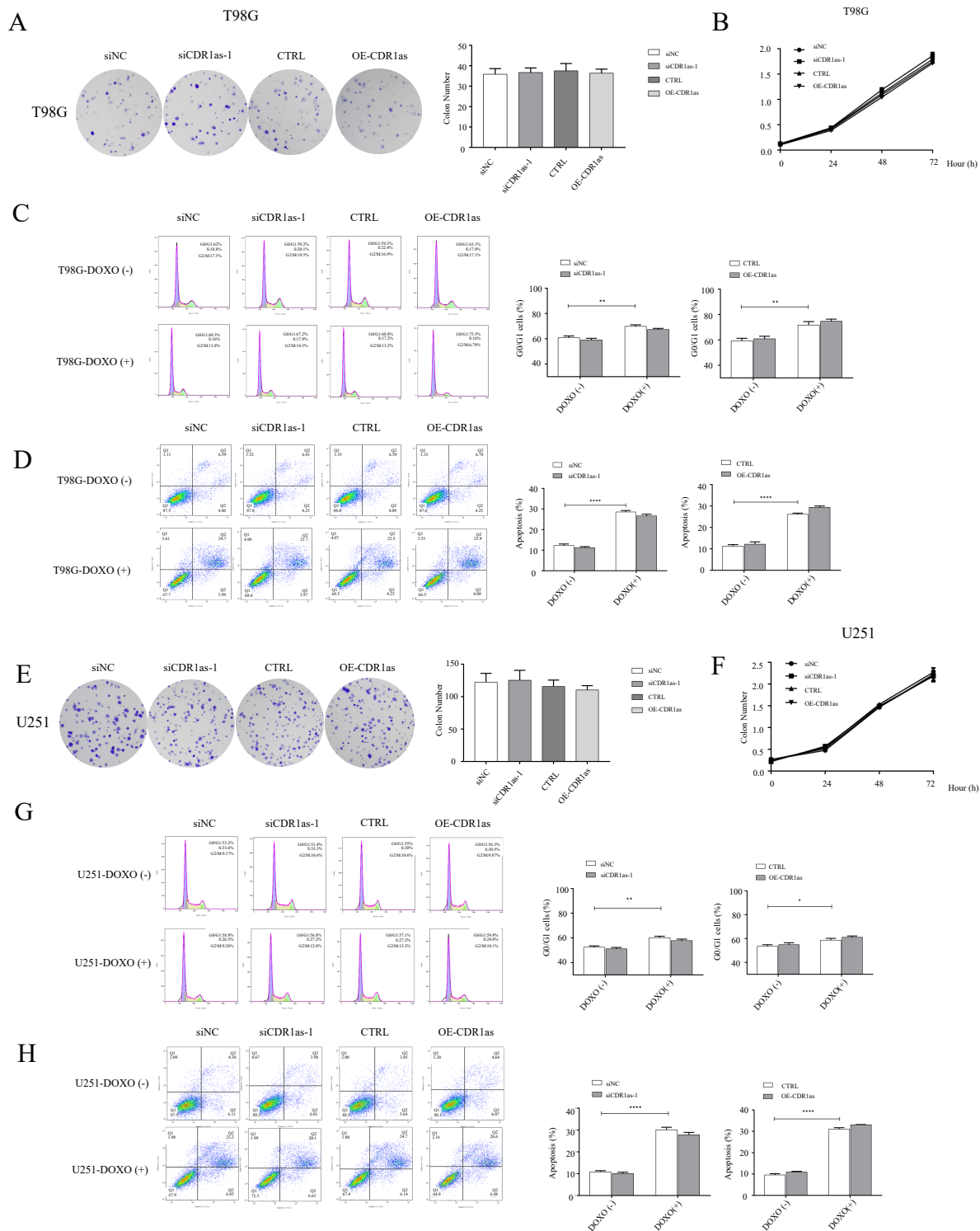

Supplement: Supplementary file 8 — Additional file 8 Figure S7. CDR1as has little effects on growth of p53-mutant GBM cells T98G and U251. A-B. Colony formation assays (A), and cell proliferation assays (B) for p53 mutant T98G cells in which CDR1as expression was manipulated. C-D. Flow cytometric cell cycle assays (C) and apoptosis assays (D) for p53 mutant T98G cells in which CDR1as expression was manipulated after 48 h treatment with DOXO or DMSO. E-F. Colony formation assays (E), and cell proliferation assays (F) for p53 mutant U251 cells in which CDR1as expression was manipulated. G-H. Flow cytometric cell cycle assays (G) and apoptosis assays (H) for p53 mutant U251 cells in which CDR1as expression was manipulated after 48 h treatment with DOXO or DMSO. *p < 0.05, **p < 0.01, ***p < 0.001, ****p < 0.0001. [file 12943_2020_1253_MOESM8_ESM.pdf]

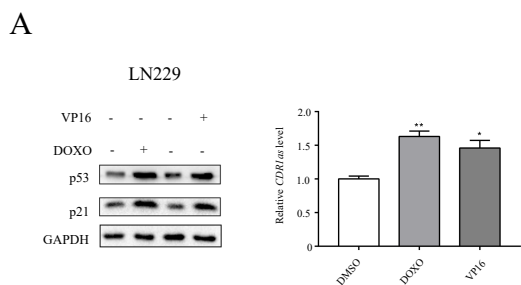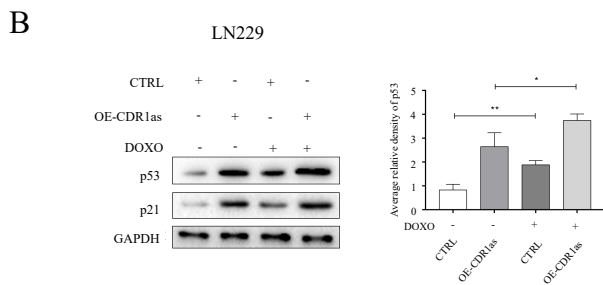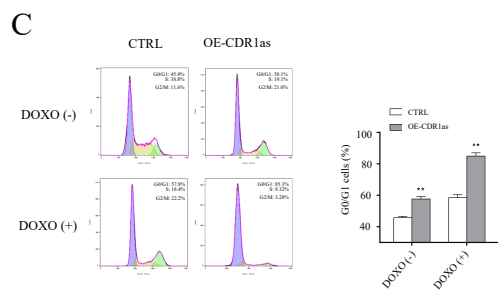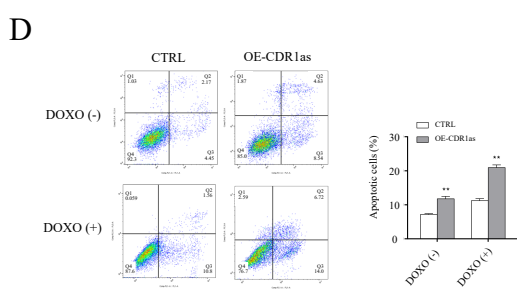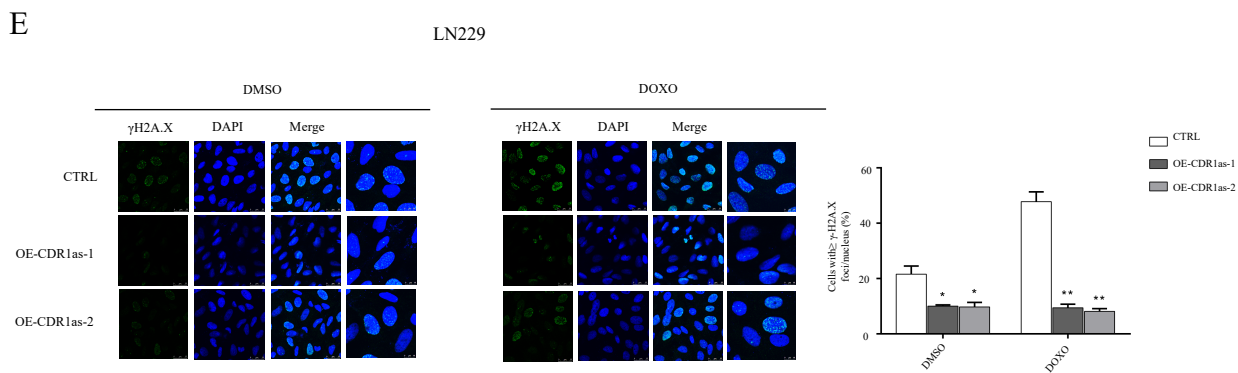

Supplement: Supplementary file 9 — Additional file 9 Figure S8. CDR1as serves as a protective machinery to preserve p53 function against DNA damage in LN229 cells. A. Western blot analysis of p53 and p21 expression (left), and RT-qPCR analysis of CDR1as expression (right) in LN229 cells after 48 h treatment of DOXO, VP16 or DMSO. B. Immunoblot of p53 and p21 (left), and densitometric analysis of p53 expression normalized to GAPDH (right) in LN229 cells transfected with plasmid encoding CDR1as or control plasmid after 48 h treatment of DOXO or DMSO. C. Flow cytometric analysis of cell cycle in LN229 cells transfected with plasmid encoding CDR1as or control plasmid after 48 h treatment of DOXO or DMSO. D. Flow cytometric analysis of apoptosis in LN229 cells transfected with plasmid encoding CDR1as or control plasmid after 48 h treatment of DOXO or DMSO. E. IF analysis of γH2A.X in LN229 cells transfected with plasmid encoding CDR1as or control plasmid after 48 h treatment of DOXO or DMSO (left); quantification of number of γH2A.X positive cells with equal or more than 10 γH2A.X foci/nucleus (right). *p < 0.05; **p < 0.01. [file 12943_2020_1253_MOESM9_ESM.pdf]

A

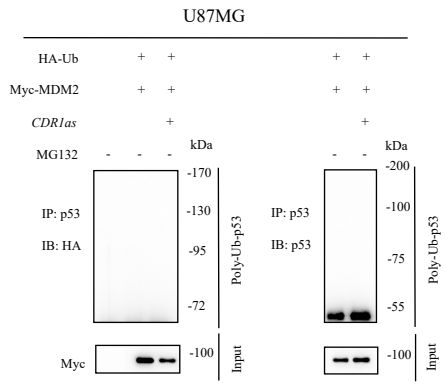

B

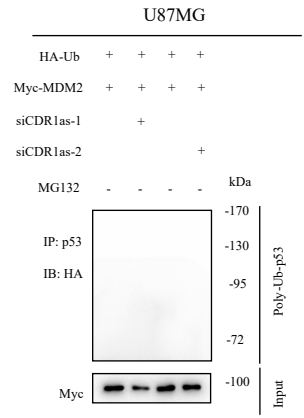

Supplement: Supplementary file 10 — Additional file 10 Figure S9. Ubiquitination of p53 in U87MG cells without MG132 treatment. A. Immunoblot of p53 ubiquitination in U87MG cells co-transfected with the plasmids encoding HA-Ub, Myc-MDM2 and CDR1as without MG132 treatment. B. Immunoblot of p53 ubiquitination in CDR1as knocked-down (or siNC treated) U87MG cells transfected with the plasmids encoding HA-Ub and Myc-MDM2 without MG132 treatment. [file 12943_2020_1253_MOESM10_ESM.pdf]
